# Stability and Efficacy of Fungicides Registered for Organic and Commercial Wheat Production in Hungary Against Fusarium Head Blight—A Comprehensive Methodology to Enhance Food Safety

**DOI:** 10.3390/toxins18030123

**Published:** 2026-03-02

**Authors:** Tamás Meszlényi, Katalin Ács, Attila Berényi, Daniel Nagy, Ákos Mesterhazy

**Affiliations:** 1 Cereal Research Non-Profit Ltd., H-6726 Szeged, Hungary; 2 Faculty of Engineering, University of Szeged, H-6724 Szeged, Hungary

**Keywords:** Fusarium head blight, wheat, fungicide use, organic fungicides, conventional fungicides, stability of fungicide effect, PCA (principal component analysis) fingerprint of fungicides, disease index, Fusarium damaged kernels, deoxynivalenol

## Abstract

Fusarium head blight (FHB) is one of the most significant diseases in wheat globally, affecting about 200 million tons of grain per year through mycotoxin contamination. Besides yield losses, mycotoxin contamination is a major concern. FHB resistance in wheat is partial and polygenic, and since the efficacy of plant protection measures is generally weak-to-moderate, an integrated approach is needed for successful control. We evaluated a more comprehensive methodology for improved protection; in this two-year study, five registered organic products and six conventional products were compared under artificial and natural infection conditions. The disease index (DI), *Fusarium*-damaged kernels (FDKs) and deoxynivalenol (DON) contamination were evaluated. The stability of the fungicides was also evaluated based on 10 epidemic conditions. The organic fungicides showed much lower efficacy than the conventional ones, although significant reductions in symptoms and DON contamination were observed. In each group, significant variability was detected. The best fungicides for DON contamination showed the lowest variance (highest stability) between 10 and 20 (Verben, Prosaro, Ascra Xpro). The organic fungicides were much less stable; the least stable showed a variance of 141 (Fusarium control: 264). The best organic fungicide was the Bordeaux mixture supported by sulfur addition (variance: 54). The DI and FDK values presented very similar trends. For the more resistant cultivar GK Pilis, the combined DON reduction exceeded 90% for all fungicides. For the most susceptible cultivar, GK Békés, the values were between 30 and 83%, respectively. High resistance to FHB and toxin contamination is the key to controlling FHB in both organic and conventional production. For efficient fungicide control, stable resistance to disease and toxin accumulation are equally required. Principal component analysis (PCA) verified the importance of considering all traits to identify the fungicidal “fingerprint” and demonstrated the differences between fungicides regardless of their organic or conventional nature. PC response differs for traits and fungicides, supporting the complex evaluation of plant and fungicide behavior. Knowledge of resistance levels, in addition to improving mycotoxin control, aids in disease forecasting and epidemic management. The results are applicable to both organic and conventional production systems. Due to the variability in resistance and fungicidal effects, there is an opportunity to improve food safety in both organic and conventional wheat production.

## 1. Introduction

*Fusarium* head blight (FHB) is one of the most destructive diseases in wheat, causing yield losses and severe mycotoxin contamination and jeopardizing human and animal health. Global wheat production is about 800 million metric tons. Of this, about 25% (200 million metric tons) is contaminated with mycotoxins according to FAO estimates [1] and 80 million tons is of preharvest (field) origin [2]. Therefore, FHB control is crucial for both organic and conventional production systems. The situation in organic production is potentially more critical as the use of conventional fungicides is forbidden. Commercial production also requires improved control measures. It is clear that organic production is a complex and sensitive system that is difficult to manage. Various factors such as the cropping system, weed control, soil microbiology, and water management interact in this system. In this study, we focused on the use of fungicides permitted in organic farming in Hungary to evaluate their efficacy against Fusarium head blight (FHB).

The main advantage of organic production is that only fungicides considered non-harmful to human and animal health can be used [3]. The papers discussed herein applied a natural induction regime. Bernhoft et al. [4] concluded that DON, ZEN, and T-2/HT-2 levels are significantly lower in organic production than in conventional production. Toxin levels in conventional production were 62% to 180% higher than in organic systems. This can be explained by crop rotation and higher organic matter content in organic soils leading to increased biological activity, as the authors state. They [3,4] also noted that certain tillage practices may increase FHB mycotoxin contamination, and some fungicides and herbicides have the same effect. In this review [4], no results from artificial inoculation were included. While resistance differences were mentioned, they could not be quantified from the data. Another paper [5] analyzed 602 samples across various environments and the resistance factor remained unassessed. These results supported the view that organic production decreases toxin contamination risk. Goral et al. [6] identified varieties under organic conditions with a lower infection severity of FHB, positing that there is less stress in the organic environment. However, the actual impact of these stresses remains experimentally unproven. The trichothecene levels were all far below set limits. Conventional fungicides are also evaluated under natural infection pressure [7,8,9]. Among low-mycotoxin-contaminated varieties, low yields and yellow rust susceptibility were observed. This agrees with our observations. Brodal et al. [10], in their review, found small or no differences in mycotoxin (DON, HT-2, T-2, ZEA) levels between the two farming systems. The weather conditions, tillage practices, year, and location played a greater role. However, they noted more controlled field trials are necessary to identify risk differences. On the other hand, Vanova et al. [11] did not find significant differences between the two production regimes. Therefore, the differentiation of genotypes and fungicides is hardly possible [12,13]. The review paper [12] did not mention the mycotoxin problem, even though they listed all the important parts of the organic/commercial aspects of the problem. *For this reason, artificial inoculation is important, as this way severe epidemics can be simulated, allowing a much better differentiation of fungicides and varieties* [9,13]. Otherwise, *evidence for variety differences and the performance of fungicides under natural inoculation remains limited.*

Severe epidemics in several years (e.g., 2010, 2015, 2019 in Hungary) have led to significant toxin problems in both systems, threatening food safety. Regional epidemics may occur more frequently (in 2–4 years out of 10). Therefore, resistance and good control is important. One approach is to evaluate fungicides registered for organic production under an artificial inoculation regime.

Which products can be used as fungicides? The answer is any product that is registered for organic use. They can be microbial products like Polyversum, used in Hungary. Sulphur products are also registered for cereals in Hungary. Many microbiological and plant products, botanical oils, etc., were tested mostly in laboratory settings. There are hopes that they might be effective [14,15]. Among them, *Trichoderma* species are often tested against various wheat diseases, including FHB [16,17,18,19,20]. In *Streptomyces* spp. [21], isolates were found that decreased symptoms and DON contamination, but this is not yet in commercial use. In spite of the large amount of information in the literature, the number of registered products is low. In another study, prothioconazole was tested in 40 trials over four years across various genotypes under natural infection conditions. The untreated controls showed a DI of 14% and 6.15 mg/kg of DON contamination (susceptible group), a DI of 10.5% and 4.1 mg/kg of DON contamination (moderately susceptible group), and a DI of 5% and 3 mg/kg of DON contamination (MR varieties). The least variability was found for the resistant group [22], as organic product have lower efficacy and higher instability and reliable information is sparse or absent. Therefore, we should concentrate on products that are available to farmers. Moreover, we should apply a methodology that is suitable for the reliable differentiation of fungicides and genotypes.

For artificial inoculation, there is data providing a wide range of epidemic scales to study. Changes in symptom severity are not always correlated with DON contamination [13]. The behavior of epidemics is characterized by DI, FDKs and DON contamination. As these often behave differently, all traits should be considered and their relationships also need clarification in organic production. The antifungal effect of fungicides is dependent on the resistance level [13]. The conclusion is that organic fungicides should be tested under an artificial inoculation regime in the field to allow the measurement of their actual antifungal effects.

The combination of resistance, fungicide technology and product choice allows for better control [23]. The question remains: how should these traits be combined to achieve maximum efficiency [22,24,25,26]? A new method was introduced to perform reliable stability analysis of data in wheat and maize resistance tests [27,28,29]. The stability of the fungicide effect across epidemics is also vital for the reliability of the fungicide response. Since 10 epidemics were analyzed, measurement of the stability or instability of the fungicide response became possible in this paper. In resistance studies, large differences in DON contamination were detected even with 1 percentage of visual ear blight [28,29]. We have data from sulfur products in a natural infection situation in the organic FHB control, which is an unsolved problem [30,31].

The withdrawal of many conventional fungicidal active agents does not affect organic plant protection as their use is not allowed. However, the combination of low organic fungicide efficacy and susceptible varieties could produce a time-bomb for organic production. The question is, what is really the case? The registered organic products have no health risk. However, the mycotoxins have a very high risk, they are 10–100-fold more toxic than the much less toxic conventional and organic products [32,33,34,35,36,37]. It should be noted that infants and young animals have tolerance levels approximately 80% lower than those of adults. This makes baby food quality a particular challenge, where the regulatory limit for DON contamination is 0.2 mg/kg. According to the cited literature [3,4,5,6,10], this limit can easily be exceeded.

The conclusion is that plant protection products that are ecologically feasible, registered for organic use, and capable of providing the required level of food safety, even for baby food, are needed. Achieving this appears to be theoretically possible, but remains a significant scientific and practical challenge.

The EU aims for a 25% organic production rate across Europe. The withdrawal of fungicides will not help conventional farming and will not improve food safety in organic production. Is it possible to find a solution that provides a significant improvement for both organic and conventional farming?

The objectives of this work were to: (1) evaluate registered organic and conventional products against FHB, considering the visual symptoms (disease index), *Fusarium*-damaged kernels (FDKs), and DON contamination; (2) assess the dependence of the fungicide effect on the aggressiveness level (using four separate isolates); (3) evaluate the combined effects of resistance and fungicide on symptoms and DON accumulation; (4) evaluate the stability of the fungicide effect across all 10 epidemics for the DI, FDK and DON contamination values; and (5) perform a PCA to identify the main underlying determining factors.

## 2. Results

### 2.1. Disease Index

Across the ten epidemic conditions, all of the fungicides decreased symptom severity (% of infected spikelets) compared to the untreated Fusarium control that was artificially inoculated and received no fungicide treatment (Table 1). The fungicides differed highly significantly, by nearly five-fold. The LSD 5% is 1.5, this is the fold of the variation width. At low epidemic severity, like in the control in 2024, all of the conventional fungicides performed similarly without significant difference. The organic-registered fungicides were less effective against FHB but significant differences were found between them. There was a seventeen-fold difference in stability between the most- and least-stable fungicides across the artificial and natural infection regimes. High stabilities of between 20 and 30 were found for Prosaro, Verben and Ascra Xpro, without significant differences in mean DI. In 2024 the *F. graminearum* isolate did not infect well, and this was also valid for its mixture. However, the *F. culmorum* isolates and their mixture had good aggressiveness, even the mean of the isolates was less in 2024. So, aggressiveness differences are more important than ecological conditions in influencing epidemic severity. Another consideration is that the mixture with the poorly reacting Fg isolate ruined the aggressiveness of the mixture in the 2024 trial, so mixing does not always seem to be a good idea.

The correlation analysis between the 10 epidemics (Table 1) shows variable results across the two years. For 2023 (highlighted in yellow), all 10 correlations are highly significant, including the control data. In 2024, however, only 3 out of 10 correlations showed significance. This demonstrates that experiments with a low infection severity are not suitable for drawing reliable conclusions about fungicide efficacy, regardless of the product tested. The not-highlighted correlations between the 2023 and 2024 data are significant between the Fc and Fcmix data; the Fg data do not correlate. This is not an effect caused by the year, but also the very low aggressiveness of the Fg inocula. The season effect is better shown by the lower natural control data in 2024, compared to the 2023 data. Additionally, there is a strong significant correlation (r = 0.98) between the variance and the mean fungicide efficacy values across epidemics. This means the better fungicides have a lower variance than the less effective ones.

The four-way ANOVA (Table 2) results showed the highest MQ values for the four traits. As the AxBxCxD interaction was not significant, the significance of all parameters was tested against the interaction term Within. The two-way interactions were all significant, but the MQ values were much lower than those found for the main effect. It is a new aspect to see the dominance or lack of dominance of the main effect over the double interaction containing the fungicide component. The main effect of variety on the AxB and AxC interactions is significant, but on a lower level (*p* = 0.05). Conversely, no significant differences were found for the fungicides or the fungicide x year interaction. The two-way interactions that did not include the fungicide factor were highly significant; however, those involving the fungicide component showed a much lower significance and their direct influence on variety or fungicide response was low. This method of analysis gives a new insight into the complicated interaction system. For us, it is important to note when a main effect is significantly different from its interaction, this is an additional proof of stability and the higher reliability of the data, in contrast to when the interaction is not significant.

**Figure 1:**
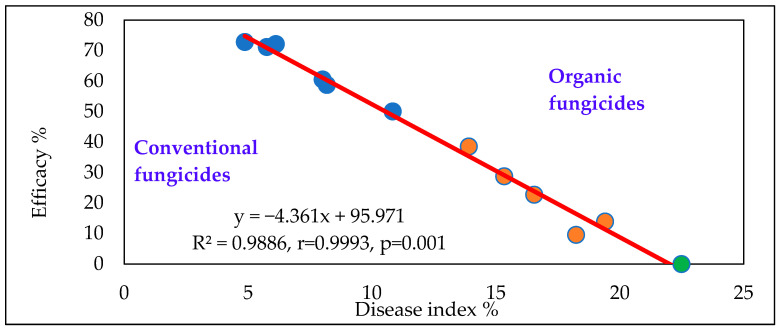
Efficacy of the organic and conventional fungicides against FHB in wheat, disease index %, across years, varieties and epidemics, Szeged, 2023–2024. Blue dots: conventional fungicide, Orange dots: organic fungicides, Green dot: Fusarium control.

The efficacy analysis of the organic and conventional fungicides (Figure 1) shows a very close negative correlation between disease index and efficacy across the 10 different epidemic conditions. The Fusarium control represents the baseline with zero efficacy, while the most effective fungicides exhibit 70–80% efficacy in reducing symptom severity. The fungicides cluster into two distinct groups, with the organic products being significantly less effective than the conventional ones. Notable significant differences were observed between these two groups.

All of the fungicides significantly reduced the disease index compared to the Fusarium control. The wheat varieties showed similar response patterns to the various fungicides, although some differences were observed (Table 3). The two most resistant cultivars responded similarly to the more effective conventional fungicides; the lowest DI values were recorded for GK Pilis and GK Szereda, and their correlations were highly significant. These two cultivars showed overlapping results until the fungicide Queen, after which DON Q showed a widening divergence compared to GK Szereda. This is a critical finding, as it indicates that a variety’s response to organic products cannot be predicted based on its performance with significantly more effective conventional fungicides. Consequently, for organic crop protection, differences in inherent resistance carry greater importance. On the other hand, the stability across the three varieties was highest for Prosaro, Verben and Ascra Xpro (values between 9.1 and 15.7). For the least effective organic products, instability was even higher than that of the Fusarium control. The stability values of the organic fungicides across the epidemics ranged between 37.5 and 81.3; in two cases, this instability exceeded the 64.9 value of the Fusarium control. Among the organic options, the Bordeaux mixture with sulfur performed the best, though it still ranked behind the least effective conventional fungicide, DON Q. The worst combination is a susceptible variety and a low-effectiveness (or non-effective) fungicide. The ratios between the two more susceptible cultivars and the experimental means were compared to that of the most resistant, GK Pilis. There is variation in the data, but significant correlations were only occasionally identified between the rates. This is very different from the highly significant correlations between the original data.

**Figure 2:**
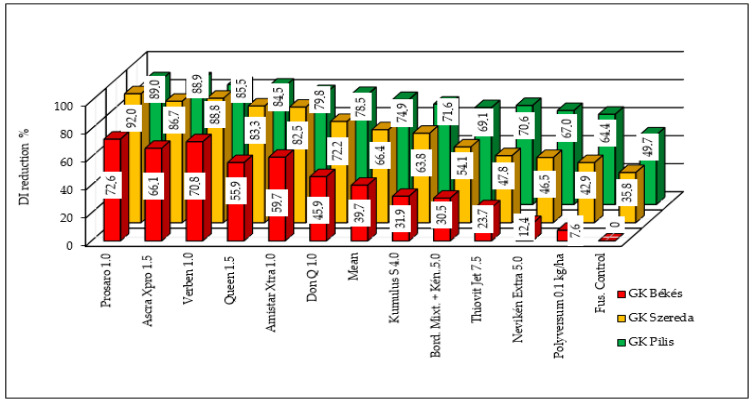
Combined reduction effect of resistance and fungicide application on DI severity in three wheat varieties treated with 11 different fungicides and one Fusarium control, 2023–2024. (Counted from Table 3, all data related to the Fusarium control of variety GK Békés).

Reduction rates were determined for each cultivar and fungicide. The fungicides showed the most stable performance with the best-performing cultivar, GK Pilis, and instability was higher in GK Szereda (Figure 2). The lowest values were produced for the susceptible GK Békés variety. The dependence of fungicide effect on resistance level is clear. This has significance when we choose the variety for fungicide tests. On a material with good resistance, nearly every fungicide will be good or very good, but with a susceptible variety even the best fungicide will provide insufficient control.

### 2.2. Fusarium-Damaged Kernels (FDKs)

The FDK data show a six-fold difference between the fungicide means (Table 4). With an LSD 5% of 1.2%, Nevikén Extra and Polyversum showed no significant effect compared to the control. This differs from the results for DI, where larger differences were recorded and a significant difference was found to the Fusarium control. The other three organic fungicides had lower FDK values than the best conventional products. The best were Verben, Ascra Xpro and Prosaro. Over the two-year period, the means for the 2023 data were more closely clustered than those in 2024, where the differences were similar to those seen for DI. Since the inocula were prepared from the same isolates, the data indicate that inocula derived from the same source do not necessarily result in the same infection severity across different years, although this can occur. The differences between the two years correspond to the disease index results in Table 1; for instance, the low aggressiveness of Fg (2024) also determined the aggressiveness of the mixture. Conversely, the Fc data were comparable to the 2023 results even though they were somewhat lower because of the dryer conditions and low performance of the *F. graminearum* inocula. The natural control data means had high agreement for the two years with no significant difference. However, the highest column mean was found with Fc alone and its mixture produced only half of this value. So, mixing was not successful to stabilize aggressiveness and this was very similar to what we found for Fg.

The correlations are lower than those observed for DI; furthermore, in contrast to the 2023 data, all of the controls except one were non-significant. In 2024, only the two *F. culmorum* data sets showed significance, while the Fg and Fgmix and control data did not. However, the tendencies and reasons were the same; for the lower results in 2024, both the lower aggressiveness and their mixing effect were responsible for the Fg results. In Fc, however, the DON contamination surpassed the 2003 data. No significant correlations were found between the artificial and natural FDK data, which is unsurprising given that the natural infection levels in the naturally infected controls were near zero.

In the ANOVA, all of the main effects were highly significant at *p* = 0.001, except for year (D), where *p* = 0.01 (Table 5). All of the analyzed interactions involving the fungicide effect were also significant. However, the magnitude of these interactions was much lower than that of the main effects; thus, the main effects remain dominant, which is a key finding. While the two-way interactions between variety, inoculum and year showed significantly higher MQ values, this did not influence the fungicide effect as much as anticipated, given the low values for the fungicide-related and other interactions (AxB, AxC, AxD). As the differences between the main effect of fungicide (A) and its three interactions are highly significant, the results are very similar to those found for DI. The variety (B) effect dominates the AxB interaction, but was non-significant in the BxD (variety x year) interaction; this agrees with the DI data. These results suggest that, within this data set, the dominance of the fungicide effect over the two-way interactions is robust and well-supported. The patterns of the dominance or non-dominance of the fungicide and variety effects correspond to the data received in DI analysis.

**Figure 3:**
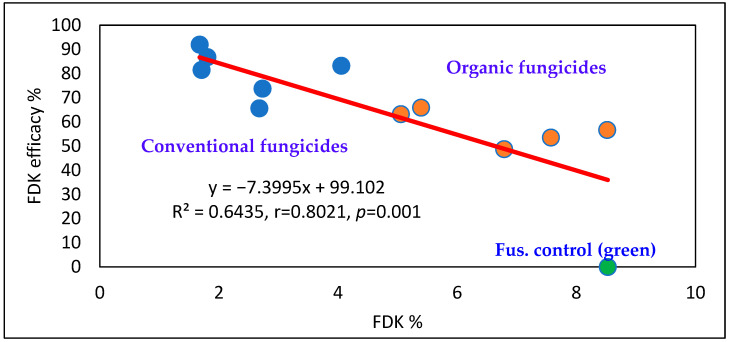
Efficacy of the organic and conventional fungicides against FHB in wheat, FDK %, across years, varieties and epidemics, Szeged, 2023–2024. Blue dot: conventional, Orange dots: organic, Green dot: Fusarium control.

Considering fungicide performance (across years, varieties and cultivars), the conventional fungicides were more effective (above 80%) than the organic alternatives (Figure 3); however, the difference between the two groups was less pronounced than that observed for visual symptoms. Organic fungicides achieved at least a 40% reduction, with a maximum of 65%.

Fungicide performance in the three genotypes was as significant as the differences in resistance (Table 6). The lowest FDK values were observed in GK Pilis; for this cultivar the FDK values for the conventional, effective fungicides were near zero and reached approximately 2% for the least effective treatment. These results suggest that the superior efficacy (2–4-fold) of conventional fungicides was consistent over organic products across all tested cultivars. The FDK value alone is not enough and therefore the toxin data will be very important. The close correlations between the responses of different varieties to the different fungicides allow the hypothesis that the fungicides act similarly in different varieties, but the extent of the infection is resistance-dependent. The rates between GK Pilis and the other varieties and experimental means show mostly highly significant correlations, but they are negative. The reason is that the FDKs in the most effective fungicide conditions had near-zero values, so the rates for these fungicides became very high.

**Figure 4:**
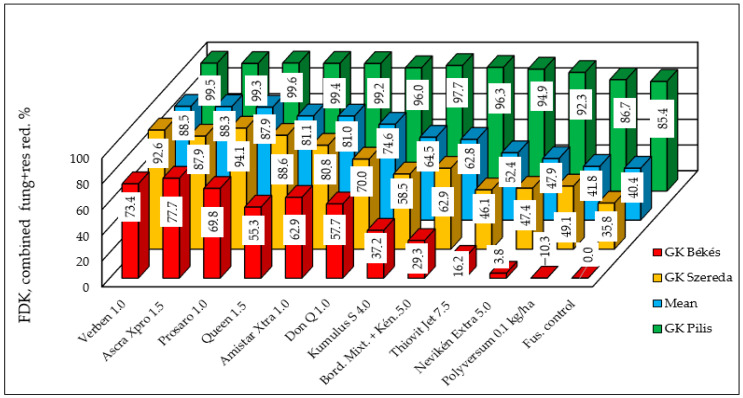
Combined reduction effect of resistance and fungicide application on FDK severity (%) in three wheat varieties treated with 11 different fungicides and one Fusarium control, 2023–2024. Counted from Table 6, all data related to the Fusarium control of variety GK Békés.

In the most resistant variety, GK Pilis, nearly all of the fungicides achieved a reduction of far over 90%. The weakest variety in terms of resistance decreased the DON contamination to 85%. The behavior of the moderately susceptible and susceptible varieties shows the same pattern with somewhat lower and much lower efficacy (Figure 4). All of the data were compared to the inoculated control of the most susceptible variety, GK Békés, with a value of 11.96% (Table 6). The combined reduction was substantially higher than that observed for the DI or FDKs. These results suggest that while the resistance level of GK Pilis may serve as protection against FDKs in organic production, higher susceptibility prevents the effective reduction of infection severity under severe epidemic conditions.

### 2.3. DON Contamination

The DON contamination data for the six conventional and five organic fungicides (Table 7) revealed highly significant differences. The toxin contamination was more severe than expected by the FDK values. Between the first four fungicides no significant difference was measured. Their stability was also high, with a variance below 20. Similarly to the DI and FDK results, the organic and conventional fungicides formed two distinct groups, although the least effective conventional and best organic fungicides showed no significant difference. The mean DON contamination in the first year—which showed substantial variance—was twice that of the second year. The DI data for Fgmix showed a high infection severity, but only a small amount of DON contamination was measured, so the FDK/DON and DI/DON relationships were different in spite of the close correlations. The highest instability was recorded for the Fusarium control (264 mg/kg); however, since even the least effective fungicide differed significantly from the control, the instability observed for Nevikén Extra lacks practical significance.

These trends in the correlations followed the pattern found for the DI and FDK values in 2023 and 2024. The correlation between stability and mean performance was highly significant (r = 0.92, *p* = 0.001), indicating that variance is a useful indicator of both the stability and the efficacy of the fungicide preparation in question. The DON contamination data from the natural infection tests did not show significant fungicide differences and this agrees with the literature data. These data support, with some differences, the information gained for DI and FDK.

The ANOVA results for the DON data (Table 8) showed a pattern similar to those observed for the DI and FDKs. The main effects are all highly significant; however, the interactions involving the fungicide effect, while still significant, were lower in magnitude. The interactions between variety, inoculum and year were strong, but their direct influence on the fungicide ranking was moderate, yet significant. The dominance of the fungicide effect relative to the two-way interactions followed the same pattern and all were highly significant, underscoring the strong influence of the fungicides. The dominance of the variety effect was significant compared to the AxB and AxC interactions, but not for the BxD (variety x year) interaction, indicating similar responses across both years, and the general trend was the same as that found for the DI and FDKs. The only difference was that the A x B interaction had a *p* = 0.05- and not a *p* = 0.001-level interaction.

**Figure 5:**
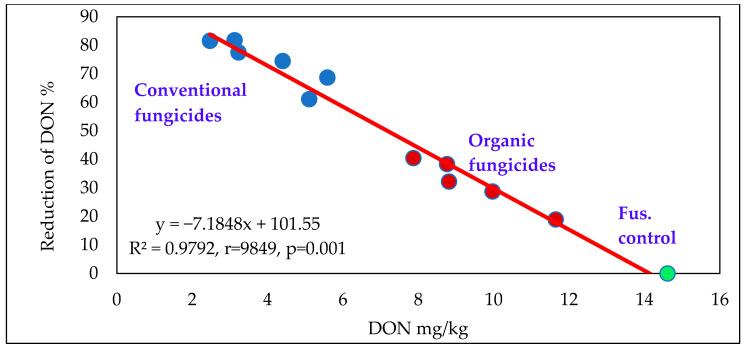
Efficacy of the fungicides against FHB in wheat; deoxynivalenol mg/kg across years, varieties and epidemics, Szeged, 2023–2024. Blue dots: conventional fungicides, Red dots: organic fungicides, Green dot: Fusarium control.

Figure 5 shows that the conventional fungicides lead to a larger DON contamination reduction than the organic products, which also have a reduction effect compared to the unprotected Fusarium control. The two groups differ significantly. For the DI and FDKs the transition was not as sharply distinct. However, even the least effective fungicides significantly reduced the DON contamination to some extent, but far less than would be necessary for effective control. We think, therefore, that the reduction of DON contamination is important, but it is successful only when its concentration can be decreased below the set limit.

The DON values of the three varieties differed significantly across the 11 fungicides and the *Fusarium* control (Table 9). The data between the fungicides on the different cultivars was very closely correlated, but this was not true for the rates between GK Pilis and other varieties. Here, no significant correlations were found. This is a strong argument that correlations alone are not enough to analyze the data; knowledge of the different response rates across different traits is essential. This varying correlation matrix could possibly be responsible for the different PC responses and fungicide distributions.

GK Békés and GK Szereda showed nearly 10-fold- and 4.4-fold-higher mean DON contamination levels, respectively, than GK Pilis. While the conventional products were suitable for GK Pilis, their efficacy was significantly higher than that of the other two varieties.

The Fusarium control data indicated that, under epidemic conditions, the DON contamination in all of the varieties can significantly exceed the official limits. However, with the application of suitable fungicides, highly and moderately resistant varieties (such as GK Pilis) can be protected much more effectively, reducing the DON levels below the official EU limit. Consequently, cultivars with less resistance than GK Pilis are not recommended in commercial production.

**Figure 6:**
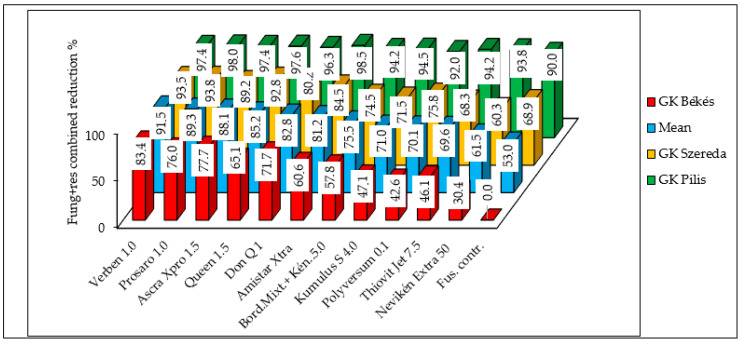
Combined reduction (%) effect of resistance and fungicide application on deoxynivalenol contamination in three wheat varieties treated with 11 different fungicides and one Fusarium control, 2023–2024. Counted from Table 9, all data related to the Fusarium control of variety GK Békés 30.09 mg/kg.

The synergistic effect of host resistance and fungicide efficacy on DON contamination reduction is of particular interest (Figure 6). In the variety GK Pilis, this combined reduction exceeded 90% for all of the fungicides, with a mean of 95.32%. This high level of resistance could permit the use of certain less effective organic fungicides, though not all. The moderately susceptible variety, GK Szereda, exhibited a mean reduction of 79.5%; however, the organic fungicides demonstrated limited efficacy at this resistance level. In contrast, the most effective conventional fungicides, Verben and Prosaro, achieved reductions exceeding 90%. Organic fungicides failed to provide sufficient protection for the susceptible variety, GK Békés, where the mean efficacy was only 54%. These data clearly demonstrate that fungicide efficacy is resistance-dependent for DON contamination as well.

The DON contamination for one percentage of DI (Table 10) shows significant differences among the genotypes across all of the fungicides, with the lowest values recorded for the most resistant variety. While no significant difference was observed among the fungicides themselves, the stability index (variance) showed five-fold differences between the fungicides; GK Pilis showed the lowest rate and the other two more susceptible cultivars had a much higher DON content for 1% DI. The data show that the more resistant cultivar (cv) has a stable DI value across fungicides, which can be important for risk assessment of varieties.

Regarding the DON concentration per 1% of FDK severity, the results show a different trend (Table 11). GK Pilis yielded mean values more than three-fold higher than the other two. This can be explained by the extremely low FDK values in GK Pilis (below 0.1%), which mathematically inflated the DON/FDK ratio for several fungicides, reaching values 9 or higher for DON content per 1% FDK. It is not an accident that the variance data are very diverse. This was independent from the organic or conventional nature of the fungicides. As this trait was very important in the resistance tests, it is misleading in the fungicide test and it is better to follow the real DON contamination level that is prescribed by the official toxin limits. The unusual data are not necessarily bad. They have their causes.

A comparison of the general means for these traits (Table 12) reveals a relatively high visual infection severity as measured by the DI, followed by the FDK values, which are approximately two-thirds lower, and followed by a significantly higher DON concentration. The LSD values are highly significant for all traits and also for *p* = 0.001, which is unusual in experimental praxis. The variance difference for the three traits is 20-fold, but in spite of the very close correlation between traits, extreme high values occur for several fungicides. The trend is clear: the conventional fungicides have a lower variance, i.e., a higher stability, and the organic fungicides are increasingly instable. The DON contaminations by % DI and FDKs do not show extreme deviations, but for FDK/DON the numbers are nearly three-fold higher, which is similar to what we found for the individual traits. The data means that between fungicides, in this respect, only small differences occurred that do not seem to be important. In the variety resistance tests these differences were large. The correlations between traits and stability are very close, but no significant correlations were found between them and the traits with DON/DI and DON/FDK. The reason for the positive and negative correlation is not clear and further research is needed. However, the correlations were closer for the FDK values and that agrees with the resistance research results data to serve as a substitute for direct DON analysis in food safety risk assessments.

In summary Table 13, the six conventional fungicides proved more effective across all parameters than those registered for organic use. Shown in the table is each variety’s three-year mean data for all traits. The most efficient group has only two of its data points in the lowest quartile (three fungicides); the data in the next group is all below the column mean, with mostly light green highlighting. The data in the better quartile of the organic fungicides is mostly above the column mean, with two data points in the best category and another two fungicides belonging to the least effective–non-effective category, together with the control group. This robust approach ensures a more reliable overall evaluation. The summary of the three traits and varieties (Table 13) presents a similar pattern across all traits. As all of the correlations are highly significant at *p* = 0.001, they are not presented in detail.

For Prosaro, all of the data belong to the most resistant group. For disease index (DI), the Verben and Ascra Xpro data all had a dark-green classification, except for one case of the DI in two cvs (cultivars). All of the data for the Fusarium control group are highlighted in orange. In terms of significance, the role of DON contamination is the highest and will therefore have the highest weight in the risk analysis. However, without the DI and FDK values the meaning of the DON contamination data is not clear.

Based on their stability (variance below 10), the first three conventional fungicides show good performance across traits and varieties. While these are the most effective fungicides, even they are unable provide complete control (with variances between 6.78 and 8.63). The stability closely correlates (r = 0.96, *p* = 0.001) with the mean data, but this does not mean that between the neighboring data there could not be large variance deviations, like between Kumulus and Polyversum or Ascra Xpro and Queen. When two fungicides have close mean data but strikingly different variances, then the lower-variance fungicide should be chosen. We also have to know whether they agree.

Principal component analysis (PCA) for disease index revealed a close relationship between GK Szereda and GK Békés, while the more resistant variety, GK Pilis, was somewhat distinct and more diverse (Supplementary Figure S1A). Regarding the fungicides (Supplementary Figure S1B), the *Fusarium* control (12) was clearly separated from all of the treated variants except fungicide 9. The other organic fungicides clustered into two loose groups (10, 11, 7, 8), as did the conventional fungicides (3, 4, 5 and 1, 2, 6), producing a more compact group with two subgroups. The PCA found Factor 1 (F1) accounted for 92.7% and Factor 2 (F2) 3% of the variance; thus, the first factor was dominant.

**Figure 7:**
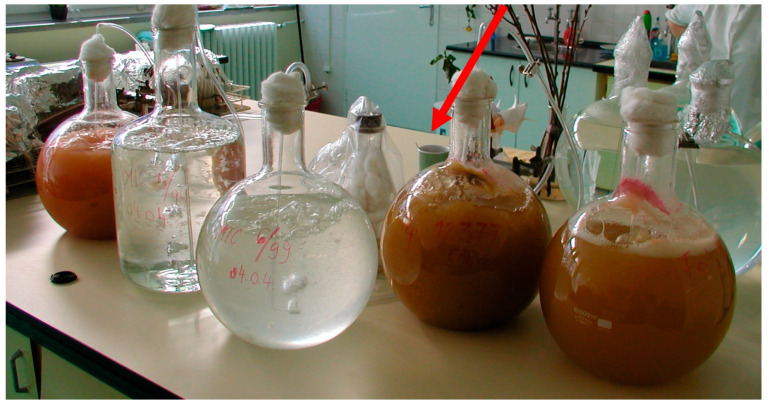
Inoculum production in 10 L glass balloons supplied with sterile ear after autoclaving at 1.30 bar for 2 h. After a week at room temperature the inoculum was ready. The compressed air came from a compressor with a 50 L store capacity; the air was sterilized by filtering with a cotton filter in the middle of the table (red arrow) and the air was transmitted from this with polyethylene pipes to the glass balloons. The image shows the suspensions ready after one-week, half-ready suspensions, and freshly inoculated balloons at the same time.

For FDK, the factors F1 and F2 accounted for 93% and 5%, respectively. The varieties (Figure 7C) follow a similar pattern to that observed for the DI, although the distance between the two more susceptible varieties is smaller and they nearly overlap with each other. The fungicides (Supplementary Figure S1D) do not tend to have clusters, but rather a looser distribution is characteristic. A clear separation between the organic and conventional fungicides was not the case; the fungicides showed a different distribution: a tight cluster was formed only by conventional fungicides 1, 2 and 3, while all of the others were scattered across the diagram.

For the DON contamination, Factors 1 and 2 accounted for 90% and 6% of the variance, respectively, showing a pattern similar to the other traits. Of the varieties (Supplementary Figure S1E), GK Pilis and GK Békés were positioned closer to each other, whereas the moderately susceptible variety, GK Szereda was more isolated. This differs from the behavior of DI and FDK. The distribution of the fungicides showed high variability (Supplementary Figure S1F) on the whole surface of the graph. The organic and conventional fungicides were not clearly separated; only two fungicides (1 and 2) formed a distinct group.

Considering the summary of all three traits (Supplementary Figure S1G), Factor 1 represented 97% and Factor 2 represented 2% of the overall variance, consistent with the results obtained from the individual trait analyses. The DI and FDKs are closely related; the DON contamination has a different position not very far from them. The fungicides (Supplementary Figure S1H) on the left side represent the organic, and those on the right side the conventional, preparates. The organic fungicides are rather far from each other and a group cannot be identified. It appears that each trait possesses unique “fingerprints” for the various fungicides studied, both across the wheat genotypes and mean data [28]. Disregarding these individual variations could lead to significant failures in disease management strategies.

## 3. Discussion

In the study we used four different isolates for inoculation, which allowed us to study a significantly higher number of epidemic situations than the one epidemic/year in all international publications. The controls without fungicides and with fungicides, and with and without inoculation, allowed comparison on a wider base than usual (one inoculum/year). In a regular two-year study with four replications, two epidemics are analyzed, and in a three-year study three cases are considered. In our case the number of epidemics was 10, three-fold higher, allowing a stability test and much higher reliability than with a two or three-epidemic data sets. In field tests every year is different. By using more isolates, we can generate additional epidemics that can provide very different results. However, looking at the severely infected columns, finding correlations between data sets and between isolates for different isolates is feasible. When the infection severity is low, nothing is sure. Therefore, we need a larger data set.

We planned the test for three years, but in 2025 the drought was severe and the plots looked to not be suitable for the tests; therefore, we had to work with two years of data, but even so the reliability of the data is much larger with 10 epidemics than the usual 2–3 years data. The other big difference is that in this study, unlike usual, a combine harvester was not used. This is a significant advantage because small and shriveled grains could be kept. Therefore, the results are scientifically feasible, as there was no loss of FDKs and DON-contaminated grains. The data are much more reliable compared to field data, where the significant loss decreases the FDK and DON values, but increases yield loss. The paper worked with a very large amount of data, for DI and FGK 3240 original data points were used, and for DON 1080.

There is data in the literature about hundreds of lab tests for organic fungicides. However, in this study we tested only those that have practical significance as they can be bought in shops. Our goal was to test these products. There were five of these products, i.e., all of the registered products in Hungary were tested. It will be another task for future research to test them with a rapid test and choose the most promising for such detailed and expensive tests. For fungicide registration this complex methodology is suggested.

### 3.1. Organic and Conventional Fungicides

In organic production, the use of conventional fungicides is prohibited. Various fungicides and other antifungal compounds have been tested under natural infection pressure [4,5,6]. Several authors [14,15,16] have evaluated the antifungal capabilities of various *Trichoderma* species; in several cases, DON levels comparable to those in conventional production were achieved. In some instances, no significant differences were found in mycotoxin contamination between the two production systems [11]. The earlier-developed artificial inoculation methodology in Szeged [7,8,13,24,25,26] was also successfully applied for organic fungicides, even they were much less effective for the most important traits than the conventional fungicides. In the PC the two groups responded differently for different traits; definite closely related compact groups were not found. However, within the groups there were also significant differences. Therefore, there was a reason to find more effective fungicides within the groups. Three of the conventional fungicides (Ascra Xpro, Prosaro, Verben) contain prothioconazole as the main active agent. The danger for commercial production is that withdrawal of this active agent can cause significant epidemics with high mycotoxin contamination. Even prothioconazole cannot effectively protect susceptible genotypes, so we cannot say that better fungicides alone can solve the problem. Organic production uses mostly conventional varieties and the resistance level of these varieties is not known; therefore, weaker fungicides mean additional toxin risk. The PCA data clearly show that the fungicides differ in FHB-influencing traits in both groups. Therefore, all traits should be considered. It seems that whatever the fungicide’s origin, its efficacy can be determined with this methodology. Of course, this leaves complete freedom to produce any product to control FHB. Close and highly significant correlations were found between the fungicides’ effects on different cultivars and traits. However, the rates between GK Pilis and the other varieties and experimental means showed highly variable characterizations (Table 3, Table 5 and Table 9). This explains the variable PC characterizations of traits and fungicides and shows that while the correlation analyses are useful, they cannot not explain everything [29]. It provides a controlled method by which fungicides’ suitability and ability to control disease symptoms and mycotoxin can be determined. The results confirm the activity of the sulfur-containing products against FHB under low infection pressure [3,4,5,6]. However, under heavy epidemic conditions, they proved insufficient, failing to provide protection when the need was greatest. It should be stated that fungicides alone, even the best ones, cannot provide full control of symptoms and mycotoxins [8,15,23]. A highly important independent variable is the resistance level of the plant.

### 3.2. Resistance and Fungicide Interaction

GK Békés, which is considered moderately susceptible [13,22,26] based on earlier tests, could not be efficiently protected by any of the organic fungicides, despite observable differences in their activity. It is not a new finding that the wheat varieties have five resistance components [27,38]. Therefore, the resistance factors influencing the DI, FDKs and DON contamination were known. However, it was not known how these differences influence fungicide efficacy. The PC tests proved that the variety distance between traits is variable, and the three traits differ from each other. Furthermore, the fungicides have different positions relative to each other in their performance on DI, FDKs and DON contamination. This is a new aspect that indicated more or less different roles in the different positions of different fungicides. This is different for all three traits and clearly shows that the use of the three traits is a scientific necessity to describe the behavior of fungicides in the different varieties. We said [29] that each variety has its own fingerprint. This is something we will have to know to develop a smart and integrated plant protection system that works better and is cheaper than the present often-used non-field-specific agronomy system.

The other aspect is the variety resistance level, which basically determines the anti-Fusarium and antitoxic effect of the fungicides. This means that this refers not only to the conventional fungicides, but it fits also for the organic fungicide products. These results support earlier findings [13,22,24,26], with the additional insight that this trend remains valid for organic fungicide products. The same phenomenon was also observed for the six conventional fungicides used for comparison. Therefore, such an effect can be hypothesized for microbial and other biological control products as well. The conclusion is that for a highly resistant cultivar in which a 95–99% reduction in DON contamination is realized, the same fungicide on a highly susceptible variety may allow a many-fold rate of the official toxin limit. For this reason, the resistance level must be known to find the best fungicide for use. We have wheat lines with significantly higher resistance levels than GK Pilis. It is a task for the future to test these materials. When we need 100% protection and the highly resistant genotype can cover, for example, 85%, a medium-effectiveness fungicide (30% efficacy) with organic qualification could secure the necessary protection and would have a 15% reserve. For the same fungicide at 10% resistance cover we would have 40% protection, and a more severe epidemic will destroy the grain.

Both conventional and organic farming face the same challenge: the low resistance level of most varieties. Considering that highly susceptible genotypes cannot be protected from mycotoxin contamination above the official limit—even by the best fungicides [9,13,22]—the required level of host resistance is higher in organic systems, where the anti-Fusarium capacity of registered organic fungicides is lower. At a reasonable high level of resistance, new products of microbial or botanical origin could perform better than currently permitted organic products used with susceptible hosts. Stricter variety registration could solve this problem, as susceptible and highly susceptible genotypes would not be permitted for cultivation in either conventional or organic farming.

### 3.3. Stability of Fungicide Effect

FHB resistance should be investigated as a polygenic trait, similar to yield, to determine its stability in wheat [29]; the results presented herein demonstrate that this approach is equally useful for fungicide testing. In the 10 epidemics the amount of infection (DON contamination) strongly varied and the ranking of the fungicides was also different; therefore, based on 2–3 data sets a correct evaluation of the amount of protective capacity and a correct ranking are not possible. It does not mean that the data in the 2–3 data sets are incorrect. Therefore, stability analysis, which has been used to estimate yielding ability for many decades, can also help in improving the reliability of the experimental results of FHB resistance [29] and fungicide evaluation, as demonstrated here. Willyerd et al. [23] presented data for Prosaro under a natural infection regime across three variety groups (MR, MS and S); the best results were achieved with the most resistant variety. This is consistent with previous findings for eight fungicides and the twelve examined in this study, justifying a generalization of the conclusions for fungicides. Similar conclusions were drawn by Amarasinghe et al. [39], who found that more resistant wheat exhibited better stability, though this stability was not explicitly quantified. For each trait, the variance for the most effective fungicides was 10-to-20-fold higher in this paper than that of the poor-performing organic fungicides. The analysis of the non-inoculated controls yielded results very similar to the findings of Vanova et al. [11]; no significant difference was found among fungicides regarding the mean of the three varieties. The inner-annual differences resulted from variations in pathogen aggressiveness and the drier weather conditions in 2024, wherein the aggressiveness differences were more important than the differences in seasonal conditions. The inclusion of ten epidemics made possible the evaluation of the stability of the fungicide effect. This represents the first attempt to apply such an approach for the three main traits. Similarly to resistance studies, fungicide evaluations require a large volume of data to provide a comprehensive picture of how fungicides perform across varied conditions.

Resistance and fungicide efficiency are interdependent. This means that on a fairly good resistant material many fungicides can be considered effective, as seen in the results for GF Pilis. In a fairly susceptible genotype, no effective fungicide could be identified, and GK Békés is not the worst one [23]. To avoid basic mistakes, the first step is the resistance test [29], this informs the omitting of susceptible cultivars or variety candidates. Then, with a fungicide test with three or four varieties with differing cultivars we can find the best fungicide(s) for a genotype that provides sufficient control [28,29,38].

### 3.4. What Should Be Used for Control of Toxigenic Fungi?

Several studies [18,19,20,21] have reported that antifungal microorganisms are effective against FHB and its associated toxins. However, such products for wheat are not registered in Hungary. It appears that current organic fungicides can only control weak epidemics; they fail to provide adequate protection during severe outbreaks. Highly susceptible cultivars remain difficult to protect even with conventional fungicides [9,13,22] under high infection pressure. Reducing both visual symptoms and toxin levels is equally important*. Control is deemed successful only when toxin contamination is reduced below EU or local safety limits* [22]. This distinction must be emphasized: while a 40–50% reduction in toxins is a positive step, if the final concentration remains 2-to-3-fold higher than the legal limit, the treatment cannot be considered a success. Nevertheless, lots with lower contamination levels might be cleaned and processed more economically than more heavily contaminated ones. Fusarium toxins are highly poisonous compounds [32] that are heat- and cooking-stable, persisting for years without degradation. In contrast, the fungicides tested are 100-to-1000-fold less toxic, degrade rapidly, and—when applied correctly—remain below safe limits at harvest [34,35,36,37]. It is mycotoxins, rather than fungicides, that pose the primary food safety challenge. While we now cultivars with resistance levels exceeding those of GK Pilis, the next objective is to identify or develop organic fungicides that can ensure food safety as effectively as the combination of moderate resistance and conventional fungicides does now. Theoretically, this would address the need to significantly reduce conventional fungicide use without jeopardizing food safety.

Without action and in the absence of sufficient resistance, high mycotoxin contamination can occur at a national level, as occurred in Hungary in 2010 and 2019, and this will cause very severe national epidemics. Therefore, combining high host resistance with organic fungicidal activity is essential. High resistance remains the most effective form of prevention; when integrated with fungicide applications, it ensures a high probability of maintaining food safety, even under epidemic conditions. As with this testing system products can be tested, the plant protection industry has a new and effective weapon to test the efficacy of the products. We suggest the introduction of testing first for the resistance of the varieties and later for the fungicides in the national survey tests.

### 3.5. How to Plan Better Plant Protection Against FHB?

As the Ministry of Agriculture supports the inclusion of resistance evaluation for toxigenic fungi in variety registration, it is hoped that the challenges of susceptibility and toxin reduction will be effectively addressed, provided that the necessary financial support is secured. We need the same action for the extended testing methodology for the registration of fungicides both for organic and conventional use. The many low-effectiveness and ineffective fungicides on the market prove that the present methodology has low efficacy and many setbacks, in spite of the slow development. Further development will come from even better resistance and more effective fungicides. These activities should be performed at the same time and for the best fungicides should be identified for each variety. This means extended research and testing activity.

Both organic and conventional varieties need significantly higher resistance than we have now. As the seed market is small, at least in Hungary, it is not economic to invest in breeding programs, and other important non-toxic diseases have not been mentioned. We think that varieties from conventional breeding programs should be tested for their suitability for organic production. This would enable us to secure a much higher rate of safe grain production even under epidemic conditions.

### 3.6. Outlook

To effectively control fungal diseases in organic farming, an integrated strategy involving resistant cultivars, biocontrol agents, and optimized agronomic techniques—such as adequate crop rotation, fertilization, and soil management—is recommended. From a holistic perspective, all genetic and agronomic practices should be optimized in the field. In this paper only DON contamination was treated. Besides DON contamination in wheat, zearalenone, nivalenol, aflatoxins, and HT-2 toxins have been observed in wheat grains, as well as in human and animal blood and urine samples [40,41,42,43]. Therefore, we need to know the resistance and toxin relations of the varieties and fungicides to be able to forecast possible plant and toxin responses and increase the efficacy of disease and toxin forecasting based mostly on meteorological data. If forecasting programs included the resistance levels of different varieties, and the antitoxic effects of the fungicides, a forecast at field level could be possible to build smart protection technology. Beyond mycotoxin issues, plant breeding must be renewed to improve adaptation to other biotic stresses; furthermore, resistance to drought, heat, and salinity is of equal significance [2]. It is now possible to significantly increase the humus content of soils, which determines microbial activity and nutrient uptake. This also improves the water-holding capacity of the soils. Furthermore, the productive soil layer should be free of compacted soil layers that inhibit rainwater infiltration. By integrating nitrogen-fixing microbes and managing pathogens and insects, a *low-toxin production system can be established,* leading to reduced losses and decreased production costs.

## 4. Conclusions

Advancements in fungicide testing technology are crucial for achieving higher food safety in both organic and conventional wheat production. The methodology described here is effective for testing all products with antifungal properties against FHB in wheat. The findings indicate that products currently registered for organic production are insufficient to provide acceptable control under epidemic conditions in susceptible varieties. Consequently, a significantly higher level of host resistance, combined with the most effective organic fungicides, is required; otherwise, acceptable food safety cannot be guaranteed. Significant investments are needed both in the breeding and plant protection industries. This approach makes it theoretically possible to reach a resistance level that allows for safe production using only organic fungicides, without relying on conventional products, that can balance to some extent the negative effects of climate change. Naturally, crop rotation and other agronomic practices must also be implemented. A methodological framework for variety registration, breeding for resistance, and measuring fungicide efficacy has been established [13,22,29] and can also be applied to the organic fungicides discussed in this paper. Among the commercial varieties, we identified GK Pilis as possessing strong resistance to FHB, achieving a 90% DON reduction, which is generally sufficient to maintain DON contamination below official limits. *Without state-controlled qualification and risk analysis of varieties and fungicides, there is little hope for improving food safety, as the fundamental data required for effective plant protection technology are absent.* Enhanced microbiological products [16,17,18,20,21] may also be helpful in developing more effective protection against FHB and its associated mycotoxins in both organic and conventional production systems. Controlling other cultivars may result in additional increases in the organic variety portfolio. A multi-toxin approach to the problem will be vital for finding long-lasting food safety solutions [17,42,43]. The occurrence of aflatoxins and HT-2 toxins far above set limits [17] also calls for active intervention. Ultimately, this is also a matter of food sovereignty that must be addressed.

## 5. Materials and Methods

The methodology followed procedures described in previous studies [13,24,25]. Reduction was calculated using the equation y = 100 − (treated/UTC) * 100 for all traits, where UTC represents the untreated Fusarium-inoculated control. The traits evaluated included DI and FDK percentage, as well as DON contamination (mg/kg). An additional control group was treated with fungicides without artificial inoculation to monitor changes in natural infection levels.

### 5.1. Plant Material

Three winter wheat (*Triticum aestivum* L.) cultivars with varying levels of resistance to Fusarium head blight were used. The awned cultivar GK Békés (susceptible) which was previously evaluated [13], was included alongside the awnless cultivars GK Szereda (moderately susceptible) and GK Pilis (moderately resistant). Since all three belong to the mid-maturing group, the treatments and inoculations could be performed on the same day.

### 5.2. Experimental Conditions and Design

Weather conditions: Both 2023 and 2024 were relatively dry; however, precipitation in May was higher in 2023 than in 2024, which accounts for the more severe infection observed in 2023 (weather data are presented in Table 14). The previous crop was oilseed rape. The experiments were conducted at the Kecskés Breeding Station of Cereal Research Ltd. in Szeged. The tested cultivars were sown in a randomized block design with three replicates, arranged in a nested factorial design (the plot size was 5 m^2^; 12 treatments were made) following the pattern described in [13]. Sowing took place between October 10 and 20 in both years. Weed control was performed in the first week of April using Solar (0.2 L/ha), Granstar (5 g/ha), and Duplosan (2 L/ha), providing 200 g cynidon-ethyl, 5 g tribenuron methyl and 600 g dichloreprop a.i. per unit, respectively, in 250 L/ha water. Aphids and cereal leaf beetles were controlled with Karate Zeon (0.2 L/ha in 250 L water/ha) as necessary. Fertilization consisted of a pre-sowing application of 60 + 60 + 60 kg/ha NPK (a.i.), followed by an additional N top-dressing of 60 kg/ha in early April. This ensured uniform flowering and spike size, providing ideal conditions for head inoculation.

### 5.3. Fungicide Treatment

The fungicides evaluated (Table 15) are used in both organic (a) and conventional (b) production. All five of the organic fungicides registered for wheat in Hungary were selected. Based on prior experience, Prosaro, Amistar Xtra, and DON Q were included, alongside three high-performance fungicides: Verben, Queen and Ascra Xpro. All of the latter contain prothioconazole, and it was of interest to determine whether they could outperform Prosaro. All fungicides were applied according to the manufacturers’ recommendations. The inclusion of all organic fungicides registered in Hungary for any fungal diseases was necessary due to the limited data regarding their efficacy against Fusarium. Furthermore, as these are the other options currently available to farmers, evaluating them against FHB is essential for improving disease control strategies in both conventional and organic production systems.

The fungicides were applied at manufacturer-recommended doses using a 1.5 L compressed-air hand sprayer. Treatments were performed at full anthesis (flowering). The spray volume was 500 L/ha; for example, if a plot received 250 mL, that was administered at 50 mL/m^2^. To ensure uniform coverage, half of the volume was sprayed in an upward direction and the other half downward, moving the sprayer in a serpentine (snake-like) pattern between the plot edges. This technique was used to target the spikes from all sides. The nozzles were positioned 20–30 cm above the spikes, angled 10–20° below the horizontal. Artificial inoculation was performed three days after the fungicide treatment to allow for sufficient system absorption. Previous tests on prothioconazole and tebuconazole degradation indicated that peak concentration is reached two days after application, followed by relatively rapid degradation. This justifies the 2–3-day interval before inoculation, ensuring that the pathogen challenge occurs when protection levels are their maximum [44]. Similar degradation data for the other active ingredients are currently available.

### 5.4. Isolates and Inoculation

The isolates originated from wheat and were identified using morphological and molecular markers [45]. The inoculum was scaled up using the bubble aeration method [46], which is capable of producing 10 L of inoculum per unit—a volume sufficient for large-scale experiments (Figure 7). The following isolates were used over the two-year period: Fg—19.42 *Fusarium graminearum*, Fc—12.37 *F. culmorum*, Fgmix—mixture of isolates 19.42 + 46.06 + G78 of *F. graminearum*, and Fcmix—mixture of isolates 12.37 + 12.51 + 52.10 of *F. culmorum*. The isolates were selected based on their aggressiveness, as determined by tests described by Mesterházy [29]; only isolates exceeding these standards were utilized. Furthermore, all of the strains had been previously screened for mycotoxin production, and only those with a high aggressiveness and a high DON-producing capacity were retained. To ensure the availability of high-quality material, 50% more inoculum was produced than required, allowing for the exclusion of any low-viability or contaminated batches. The inoculum was used undiluted. On the day prior to application, the inoculum was homogenized in a blender, and the spikes were sprayed the following morning.

**Figure 8:**
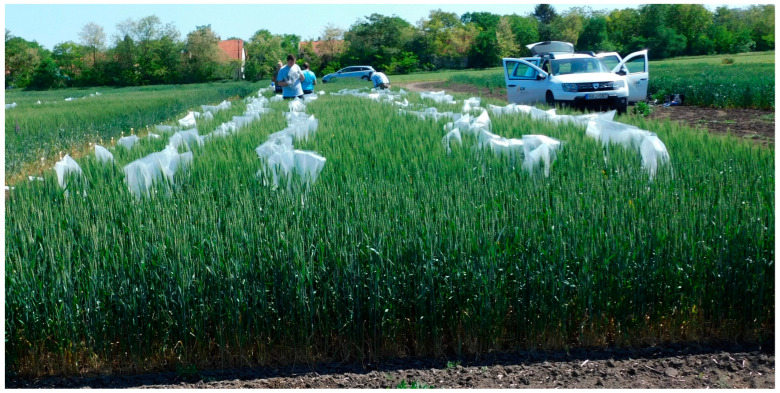
Arrangement of the fungicide tests. In the middle is variety 2, on the right variety 1, and on the left variety 3. In the middle bed from left to right: control, inocula 4, 3, 2, and 1 in three replicates (see Figure 9). No. of inoculated groups of heads: 3240 for the two years. This picture is from 2012 but reflects the experiments performed in this study in 2023–2024.

**Figure 9:**
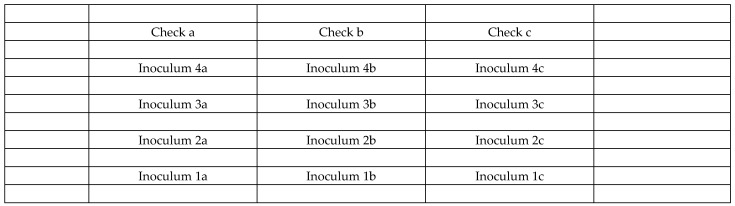
Positioning of the wheat head bunches (groups of about 15 heads) in a 1 × 5 m size plot. A rand was left to control rand effect and secure more uniform ear size and flowering time.

Inoculation was performed using the same type of hand sprayer two days after the fungicide treatment. Spikes were grouped into bunches of approximately 15; a distance of 60–70 cm was maintained between different isolates, while 20–30 cm was left between bunches of the same isolate. Each bunch was sprayed from all sides with approximately 15 mL of inoculum. For each isolate, three replicate bunches were treated and subsequently enclosed in polyethylene bags for 48 hrs. (as shown in Figure 8) [46]. To prevent cross-contamination, control bunches were established at the right end before applying the inoculum. All of the bunches were positioned 20–30 cm from the plot edges to avoid edge effects. Thereafter, the inoculations were made with the four inocula. Figure 9 illustrates the layout of isolates 1–4 and the non-inoculated control bunches within a 5m^2^ plot and the picture of the inoculated plots (Figure 8).

Disease severity was first assessed 21 days post-inoculation, following the methodology of Bai and Sharen [47]. In 2025, assessment continued until the spikes lost their green color (senescence). The percentage of visually infected spikelets was estimated for each 15-head bunch. After harvest, threshing was performed using a Wintersteiger LD180 laboratory thresher (Wintersteiger AG, Ried, Austria) at a low speed, without air-assisted cleaning to ensure the retention of all small, infected or shriveled grains. Subsequent fine cleaning was conducted with an Ets Plaut-Aubry air separator (41290 Conan-Oucques, France) with the airflow precisely regulated to retain light kernels. For FDK analysis, only grains exhibiting characteristic white or pinkish discoloration were recorded. Small, shriveled grains without visual symptoms were excluded, as their condition may result in vascular occlusion (blockage of the tracheae) and nutrient deficiency rather than direct infection. This rigorous protocol ensured the reliability of the resulting FDK and DON contamination data.

### 5.5. DON Analyses

The grain yield from the three bunches per plot was pooled. From this composite sample, 6 g was taken and milled using a Perten Laboratory Mill 3310 (Perten Instruments, 126 53 Hägersten, Sweden). A 1 g aliquot of the resulting flour was then used for DON analysis. The measurements were performed using an Agilent Infinity 1260 HPLC instrument (Agilent Technologies Santa Clara, CA, USA).

The methanol (HPLC grade, ≥99.9%) and acetonitrile (HPLC grade, ≥99.9%) were sourced from Sigma-Aldrich (St Louis, MO, USA). Ultrapure water was produced using an Adrona Connect system (Riga, Latvia). The certified standard solutions were bought from Sigma Aldrich (St Louis, MO, USA). Stock solutions were subsequently diluted with an acetonitrile: water = 20:80 mixture to obtain the required working solutions for calibration.

All of the solutions were stored at −20 °C in glass vials before use. The detailed methodology is described in Gyorgy et al. [48].

### 5.6. Statistics

Evaluation was performed separately for the DI and FDK traits. Initially, 3240 individual data points were recorded; however, the three measurements within each plot for a given isolate were averaged prior to the ANOVA. Consequently, the three replicates in the ANOVA represent plot-level replication, emphasizing the robust data foundation of the study. For DON analysis, the three sub-samples per isolate were measured individually within each plot and subsequently pooled; the ANOVA was performed using fixed effects based on 1080 data points. Thus, all three data tables contained an identical number of observations. Four-way ANOVAs were conducted following the methods of Sváb [49] and Weber [50]. This approach allowed for the determination of whether main effects predominated over two-way interactions. The MS (A) was divided by the MS of the respective interaction. This served as the F-test, with the interaction term treated as the error term (Within) for the analysis. The resulting F-values were compared against critical values from Fisher and Yates tables [49,50]. The discussion focuses primarily on interactions involving Factor A (fungicide) and Factor B (variety). PCA (principal component analysis) was performed using Statistica 13.0 software (Informer Technologies Inc., www.informer.com, Tibco, Santa Clara, CA, USA, accessed on 12 February 2022) [51]. The stability tests were conducted in Microsoft Excel (Microsoft Office 365) via one-way ANOVA as the variance was deemed suitable for this purpose [29].

Stability in terms of fungicides describes the behavior of fungicides under different epidemic conditions. Stability can be counted with the original data or with the variety ranks. Both have their advantages and disadvantages. In this paper we used the original data as they are more informative. The ranks can be interesting later, but cannot replace the original data. In a study of wheat resistance two evaluation methods were compared [29]. The Eberhart and Russel [52] method is based on a linear regression model wherein the means of 30 experiments are compared with the data of the individual varieties. The stability index was the b value of the y = a + bx. We applied this method first in 1995 [13]. In a recent paper this was compared with the results of a polynomial function performed for all three traits in four independent test series and a comparison was made between these stabilities with the measured resistance traits. The variance values were also computed based on the Excel one-way ANOVA’s variance data, and the variances often had closer correlations with the resistance traits than Eberhart and Russel’s “b” values did. The explanation is that the regressions were not always linear; we often received much higher stability with the polynomial regression data and therefore the variance that used only the original data was not influenced negatively by the mistake of the dogma of linearity. This was the reason that variance was used for the stability evaluation in this paper as the inbuilt mistake of linearity could be avoided. Additionally, its calculation is very simple. The general means of GK Szereda and GK Békés as well the means for each trait were divided by the data of the most resistant variety GK Pilis. This rate was necessary to see how different or uniform the individual fungicide responses would be.

## Figures and Tables

**Table 1:** Organic and conventional fungicides against FHB in wheat; disease index (%) means across three differently resistant varieties, 2023–2024.

Fungicide L/ha	Fg^X^	Fc	Fgmix	Fcmix	Ctrl	Fg	Fc	Fgmix	Fcmix	Ctrl	Mean	Stability Variance
	2023 Disease Index %					2024 Disease Index %						
^a^ Prosaro 1.0 ^a^	11.9	10.7	10.6	4.7	1.9	0.4	5.9	0.4	2.1	0.0	4.9	21.9
Verben 1.0	15.1	11.1	11.3	6.1	1.5	0.2	6.7	0.0	5.6	0.0	5.8	29.2
Ascra Xpro 1.5	12.3	12.2	9.4	5.2	1.3	1.6	11.4	0.3	7.5	0.0	6.1	25.9
Queen 1.5	15.5	15.9	14.1	8.1	2.3	0.4	14.6	0.7	8.3	0.0	8.0	45.3
Amistar Xtra 1.0	20.6	15.9	13.8	8.3	2.6	0.0	14.6	0.3	5.7	0.0	8.2	57.6
Don Q 1,0	24.3	18.7	14.4	10.7	2.7	0.9	26.1	0.4	10.1	0.0	10.8	97.7
^*b*^ *Kumulus S 4.0* ^b^	28.1	26.9	21.3	17.1	2.6	1.2	30.9	0.7	10.1	0.1	13.9	155.2
*Bordeaux mixt. + Kén Neo Sc 5.0*	31.4	30.1	23.4	19.6	3.6	0.2	27.1	0.5	17.4	0.0	15.3	169.3
*Thiovit Jet 7.5*	30.3	34.4	25.9	18.6	4.1	0.5	30.9	1.2	19.5	0.0	16.5	192.7
*Nevikén Extra 5.0*	31.2	30.0	28.1	20.7	5.9	1.5	38.6	0.7	25.6	0.0	18.2	216.9
*Polyversum 0.1 kg/ha*	32.9	33.5	28.5	17.6	4.0	0.1	45.0	0.7	31.8	0.0	19.4	290.1
Fusarium control	40.7	36.5	30.0	23.5	4.7	2.3	52.9	1.0	33.2	0.0	22.5	368.2
Mean	24.5	23.0	19.2	13.4	3.1	0.8	25.4	0.6	14.8	0.0	12.5	
LSD 5%	4.9	4.9	4.9	4.9	4.9	4.9	4.9	4.9	4.9	4.9	1.5	
LSD 10%					4.1							
Correlation		Fg	Fc	Fgmix	Fcmix	Ctrl	Fg	Fc	Fgmix	Fcmix	Ctrl	Mean
Fc		0.96 ***										
Fgmix		0.95 ***	0.97 ***									
Fcmix		0.97 ***	0.97 ***	0.96 ***								
Ctrl		0.84 **	0.85 **	0.91 ***	0.88 **							
Fg		0.35	0.29	0.29	0.38	0.31						
Fc		0.95 ***	0.92 ***	0.93 ***	0.92 ***	0.83 **	0.47					
Fgmix		0.67 *	0.79 **	0.75 **	0.72 **	0.69 *	0.33	0.70 *				
Fcmix		0.88 ***	0.89 ***	0.93 ***	0.86 **	0.85 **	0.38	0.94 ***	0.64 *			
Ctrl		0.12	0.12	0.08	0.17	−0.12	0.19	0.11	0.10	−0.136		
Mean		0.97 ***	0.97 ***	0.98 ***	0.96 ***	0.88 ***	0.39	0.98 ***	0.73 *	0.95 ***	0.07	
Var		0.95 ***	0.93 ***	0.95 ***	0.92 ***	0.83 **	0.42	0.97 ***	0.69 *	0.96 ***	0.04	0.98 ***

*** *p* = 0.001, ** *p* = 0.01, * *p* = 0.05, ^X^ Fg = *F, graminearum*, Fc = *F. culmorum*, Fgmix = mixture of two *F. graminearum* isolates, Fcmix = mixture of two *F. culmorum* isolates. Yellow highlight: correlations for epidemics in 2023 and 2024. ^a^ nonorganic fungicide, ^b^ *organic fungicides*.

**Table 2:** Four-way ANOVA for the fungicide test; disease index data with organic and conventional fungicides against FHB in wheat, 2023–2024.

Traits		SQ	df	MQ	F	LSD 5%
A, fungicide		35,348.92	11	3213.54	116.01 ***	1.54
B, variety		23,912.71	2	11,956.35	431.64 ***	0.77
C, inoculum		57,371.53	4	14,342.88	517.79 ***	0.99
D, year		18,781.26	1	18,781.26	678.02 ***	0.63
AxB		3904.02	22	177.46	6.41 ***	
AxC		16,586.53	44	376.97	13.61 ***	
AxD		1706.13	11	155.10	5.60 ***	
BxC		16,159.11	8	2019.89	72.92 ***	
BxD		3238.14	2	1619.07	58.45 ***	
CxD		31,396.73	4	7849.18	283.36 ***	
AxBxC		4278.06	88	48.61	1.76 **	
AxBxD		834.58	22	37.94	1.37 *	
AxCxD		9416.19	44	214.00	7.73 ***	4.1 (*p* = 0.1)
BxCxD		6244.46	8	624.45	116.01 ***	
AxBxCxD		2748.84	88	31.24	1.13 ns	
Within		19,936.88	720	27.69		
Total		254,428.36	1079			
Significance between main effects and two-way interactions						
Trait	df Main effects	Interaction	df Int.	F	LSD 5% table	LSD 0.1% table
Fungicide A	11	AxB	22	18.1 ***	2.3	3.85
	11	AxC	44	8.6 ***	2.01	3.12
	11	AxD	11	20.9 ***	2.52	5.27
Variety B	2	AxB	2	67.3 *	9.55	999
	2	BxC	8	5.9 *	4.46	18.5
	2	BxD	2	7.4	9.55	999

*** *p* = 0.001, ** *p* = 0.01, * *p* = 0.05.

**Table 3:** FHB mean data of differently resistant wheat varieties across four isolates and two years measured by disease index (DI%), 2023–2024.

Fungicide	GK Pilis	GK Szereda	GK Békés	Mean	Sze/Pi	Bé/Pi	Mean/Pi	Variance
^a^ Prosaro	3.47	2.52	8.62	4.87	0.73	2.49	1.40	10.8
Verben	4.54	3.53	9.19	5.75	0.78	2.02	1.27	9.1
Ascra Xpro	3.49	4.19	10.67	6.12	1.20	3.06	1.75	15.7
Queen	4.89	5.26	13.87	8.01	1.08	2.84	1.64	25.8
Amistar Xtra	6.36	5.51	12.66	8.18	0.87	1.99	1.29	15.3
Don Q 1	6.76	8.73	17.02	10.84	1.29	2.52	1.60	29.7
Kumulus S	8.92	11.37	21.42	13.90	1.27	2.40	1.56	43.9
^b^ Bord. mixt. + Kén Neo SC	9.70	14.44	21.85	15.33	1.49	2.25	1.58	37.5
Thiovit Jet	9.23	16.40	24.00	16.54	1.78	2.60	1.79	54.6
Nevikén Extra	10.36	16.81	27.53	18.23	1.62	2.66	1.76	75.2
Polyversum	11.20	17.96	29.06	19.40	1.60	2.59	1.73	81.3
Fungicide control	15.83	20.19	31.44	22.49	1.28	1.99	1.42	64.9
Mean	7.89	10.58	18.94	12.47				
LSD5% Fungicide				1.54				
LSD5% Variety				0.77				
Correlations	GK Pilis	GK Szereda	GK Békés	Mean	Sze/Pi	Bé/Pi	Mean/Pi	
GK Szereda	0.947 ***							
GK Békés	0.952 ***	0.988 ***						
Mean	0.970 ***	0.994 ***	0.996 ***					
Sze/Pi	0.625	0.828 ***	0.800 **	0.782 **				
Bé/Pi	−0.353	−0.128	−0.098	−0.162	0.326			
Mean/Pi	0.170	0.433	0.434	0.383	0.815 **	0.812 **		

*** *p* = 0.001, ** *p* = 0.01, ^a^ conventional fungicide, ^b^ organic fungicides, Sze = Szereda, Pi = Pilis, Bé = Békés.

**Table 4:** Organic and conventional fungicides against FHB in wheat, FDK (%), means across three differently resistant varieties, 2023–2024.

Fungicides L or kg/ha	Fg^x^	Fc	Fgmix	Fcmix	Ctrl	Fg	Fc	Fgmix	Fcmix	Ctrl	Mean	Stability Variance
	2023 FDK %					2024 FDK %						
^a^ Verben 1.0	4.87	2.02	0.46	2.76	0.11	0.06	2.02	0.03	1.40	0.00	1.37	2.54
Ascra Xpro 1.5	2.61	1.87	0.56	1.78	0.11	0.18	4.23	0.16	2.44	0.06	1.40	2.01
Prosaro 1.0	4.72	2.96	0.43	3.19	0.06	0.08	2.06	0.16	0.87	0.00	1.45	2.82
Queen 1.5	4.39	4.72	1.89	3.33	0.11	0.00	6.51	0.06	1.57	0.06	2.26	5.55
Amistar Xtra 1.0	5.13	4.44	0.81	2.54	0.11	0.01	6.56	0.22	2.20	0.74	2.28	5.53
DON Q 1.0	6.96	4.89	1.30	4.10	0.11	0.15	9.69	0.04	2.80	0.37	3.04	11.14
^*b*^ *Kumulus S 4.0*	8.15	8.07	3.74	6.09	0.22	1.61	11.13	0.34	3.12	0.02	4.25	15.38
*Bord. Mixt. + Kén.5.0*	7.80	8.17	2.19	6.69	0.00	0.06	14.01	0.35	5.21	0.00	4.45	22.53
*Thiovit Jet 7.5*	12.72	11.50	2.85	6.59	0.17	0.08	15.17	0.84	6.67	0.34	5.69	33.01
*Nevikén Extra 5.0*	11.37	7.93	4.83	8.13	0.56	0.07	17.85	1.39	10.04	0.19	6.24	34.94
*Polyversum 0.1*	9.89	9.78	3.46	8.45	0.28	0.04	23.33	0.63	13.63	0.11	6.96	58.11
Fusarium control	12.50	7.94	3.35	6.54	0.00	0.27	28.37	0.36	11.89	0.08	7.13	79.04
Mean	7.59	6.19	2.16	5.02	0.15	0.22	11.74	0.38	5.15	0.16	3.88	22.72
LSD 5%	5.16	5.16	5.16	5.16	5.16	5.16	5.16	5.16	5.16	5.16	1.20	
Correlation		Fg	Fc	Fgmix	Fcmix	Ctrl	Fg	Fc	Fgmix	Fcmix	Ctrl	Mean
Fc		0.88 ***										
Fgmix		0.82 **	0.81 **									
Fcmix		0.87 ***	0.89 ***	0.90 ***								
Ctrl		0.35	0.31	0.64 *	0.50							
Fg		0.09	0.18	0.34	0.15	0.10						
Fc		0.86 ***	0.78 **	0.79 **	0.84 ***	0.23	0.05					
Fgmix		0.73 **	0.67 *	0.79 **-	0.76 **	0.79 **	−0.04	0.56				
Fcmix		0.80 **	0.72 **	0.76 **	0.84 ***	0.40	−0.09	0.94 ***	0.66 *			
Ctrl		0.70 *	0.07	−0.11	−0.14	0.09	−0.23	−0.02	0.10	−0.05		
Mean		0.93 ***	0.88 ***	0.88 ***	0.93 ***	0.39	0.10	0.96 ***	0.71 **	0.93 ***	−0.01	
Var		0.83 ***	0.70 *	0.69 *	0.77 **	0.15	−0.01	0.97 ***	0.49	0.94 ***	−0.09	0.92 ***

*** *p* = 0.001, ** *p* = 0.01, * *p* = 0.05, ^X^ Fg = *F, graminearum*, Fc = *F. culmorum*, Fgmix = mixture of two *F. graminearum* isolates, Fcmix = mixture of two *F. culmorum* isolates, Ctrl = control. Yellow highlight: correlations between epidemics in 2022 and 2023. ^a^ conventional fungicide, ^b^ *organic fungicides*.

**Table 5:** Four-way ANOVA for the fungicide test; FDK data with organic and conventional fungicides against FHB in wheat, 2023–2024.

Trait		SQ	df	MQ	F	LSD 5%
A, Fungicide		4821.94	11	438.36	24.77 ***	1.20
B, Variety		8100.62	2	4050.31	228.88 ***	0.60
C, Inoculum		10,370.32	4	2592.58	146.50 ***	0.77
D, Year		128.41	1	128.41	7.26 **	0.49
AxB		1868.56	22	84.93	4.80 ***	
AxC		4017.94	44	91.32	5.16 ***	
AxD		395.93	11	35.99	2.03 **	
BxC		4696.53	8	587.07	33.17 ***	
BxD		1497.28	2	748.64	42.30 ***	
CxD		4644.82	4	1161.20	65.62 ***	
AxBxC		1948.24	88	22.14	1.25 ns	
AxBxD		576.31	22	26.20	1.48 ns	
AxCxD		2523.07	44	57.34	3.24 ***	
BxCxD		6244.46	8	780.56	44.11 ***	
AxBxCxD		2748.84	88	31.24	1.77 ns	
Within		12,124.32	720	17.70		
Total		61,454.40	1079			
Significance between main effects and two-way interactions						
Trait	df Main effects	Interaction	df Int.	F	LSD 5% table	LSD 0.1% table
Fungicide A	11	AxB	22	5.1 ***	2.3	3.85
	11	AxC	44	4.8 ***	2.01	3.12
	11	AxD	11	12.1 ***	2.52	5.27
Variety B	2	AxB	2	47.6 *	9.55	999
	2	BxC	8	6.9 *	4.46	18.5
	2	BxD	2	5.4 ns	9.55	999

*** *p* = 0.001, ** *p* = 0.01, * *p* = 0.05, ns = non-significant.

**Table 6:** FHB mean data of differently resistant wheat varieties across four isolates and two years measured by FDK %, 2023–2024.

Fungicide	GK Pilis	GK Szereda	GK Békés	Mean	Sze/Pi	Be/Pi	Mean/Pi	Variance
^a^ Prosaro	0.04	0.70	3.61	0.37	15.81	81.23	8.41	3.60
Verben	0.06	0.88	3.18	0.47	15.84	57.24	8.42	2.62
Queen	0.08	1.37	5.35	0.72	17.86	69.74	9.43	7.54
Ascra Xpro	0.09	1.45	2.66	0.77	16.48	30.34	8.74	1.66
Amistar	0.09	2.29	4.05	1.19	24.43	43.09	12.72	3.92
Don Q	0.48	3.58	5.06	2.03	7.43	10.48	4.21	5.45
*^b^* *Bord. Mixt + Kén Neo SC5.0 5.0c*	0.45	4.44	8.46	2.44	9.93	18.93	5.47	16.04
*Kumulus S*	0.27	4.97	7.51	2.62	18.10	27.36	9.55	13.47
*Thiovit Jet*	0.61	6.45	10.02	3.53	10.51	16.34	5.76	22.55
*Nevikén Extra*	0.92	6.29	11.50	3.60	6.85	12.53	3.93	28.00
*Polyversum*	1.59	6.09	13.20	3.84	3.82	8.28	2.41	34.22
Fusarium control	1.75	7.68	11.96	4.71	4.38	6.83	2.69	26.28
Mean	0.54	3.85	7.21	2.19	12.62	31.86	6.81	13.78
LSD5% Fungicide				1.20				
LSD5% Variety				0.60				
Correlation	GK Pilis	GK Szereda	GK Békés	Mean	Sze/Pi	Be/Pi	Mean/Pi	
GK Szereda	0.846 ***							
GK Békés	0.891 ***	0.931 ***						
Mean	0.897 ***	0.994 ***	0.946 ***					
Sze/Pi	−0.836 ***	−0.720 **	−0.767 **	−0.761 **				
Be/Pi	−0.712 **	−0.843 ***	−0.704 *	−0.838 ***	0.696 *			
Mean/Pi	−0.836 ***	−0.720 **	−0.767 **	−0.761 ***	1.000 ***	0.696 *		
Variance	0.883 ***	0.899 ***	0.992 ***	0.918 ***	−0.761 **	−0.679 *	−0.761 *	

*** *p* = 0.001, ** *p* = 0.01, * *p* = 0.05. ^a^ conventional fungicide, ^b^ organic fungicide, Sze = Szereda, Pi = Pilis, Bé = Békés.

**Table 7:** Organic and conventional fungicides against FHB in wheat; DON contamination mg/kg; means across three differently resistant varieties, 2023–2024.

Fungicide L or kg/ha	Fg	Fc	Fgmix	Fcmix	Ctrl	Fg	Fc	Fgmix	Fcmix	Ctrl	Mean	Var.
			**2023**					**2024**				
^a^ Verben 1.0	10.25	1.63	0.60	6.19	0.28	0.59	3.45	0.33	1.95	0.41	2.57	10.7
Prosaro 1.0	12.52	4.74	1.44	6.69	0.36	0.43	2.92	0.48	2.08	0.65	3.23	15.0
Ascra Xpro 1.5	11.83	3.99	1.13	7.26	0.20	3.11	4.84	0.75	1.95	0.70	3.58	13.3
Queen 1.5	10.32	5.80	2.50	11.28	0.18	0.65	8.73	0.66	4.30	0.16	4.46	18.9
Don Q 1.0	13.54	4.73	2.04	9.92	0.28	0.71	12.82	0.76	5.99	1.11	5.19	26.8
Amistar Xtra 1.0	18.81	8.92	3.37	10.10	0.75	0.66	9.05	0.63	4.12	0.25	5.67	36.2
*^b^ Bord. Mixt. + Kén.5.0*	14.04	8.17	4.51	17.58	0.83	0.57	19.57	0.45	7.31	0.69	7.37	54.2
*Kumulus S 4.0*	28.22	12.45	4.37	21.02	0.53	1.05	11.92	0.63	6.47	0.50	8.72	93.1
*Polyversum 0.1*	18.40	9.46	6.83	15.52	0.39	2.49	21.31	1.30	13.48	0.69	8.99	61.3
*Thiovit Jet 7.5*	25.62	19.58	6.23	14.50	1.01	0.60	14.56	0.52	8.36	0.61	9.16	81.5
*Nevikén Extra 5.0*	35.29	13.13	5.44	21.68	0.77	0.65	22.44	1.38	14.58	0.46	11.58	143.9
Fusarium control	42.87	19.42	6.99	19.42	0.22	0.91	40.56	1.16	9.51	0.47	14.15	264.1
Mean	20.14	9.34	3.79	13.43	0.48	1.04	14.35	0.75	6.68	0.56	7.06	68.29
LSD 5%	7.46	7.46	7.46	7.46	7.46	7.46	7.46	7.46	7.46	7.46	2.36	
Correlations	Fg	Fc	Fgmix	Fcmix	Ctrl	Fg	Fc	Fgmix	Fcmix	Ctrl	Mean	
Fc	0.87 ***											
Fgmix	0.76 **	0.87 ***										
Fcmix	0.79 **	0.73 **	0.82 **									
Ctrl	0.27	0.49	0.45	0.42								
Fg	−0.13	−0.15	0.02	−0.10	−0.36							
Fc	0.80 **	0.73 **	0.83 ***	0.74 **	0.10	−0.02						
Fgmix	0.61 *	0.41	0.61 *	0.56	−0.09	0.36	0.67 *					
Fcmix	0.65 *	0.62 *	0.858 ***	0.79 **	0.37	0.07	0.74 **	0.81 **				
Ctrl	−0.15	−0.13	−0.04	−0.08	−0.05	0.24	0.04	0.06	0.10			
Mean	0.12	0.11	0.03	−0.13	−0.32	−0.28	0.13	−0.09	−0.05	−0.28		
Variance	0.01	−0.07	−0.17	−0.27	−0.31	−0.25	−0.01	−0.22	−0.21	−0.26	0.92 ***	

*** *p* = 0.001, ** *p* = 0.01, * *p* = 0.05, Fg = *F, graminearum*, Fc = *F. culmorum*, Fgmix = mixture of two *F. graminearum* isolates, Fcmix = mixture of two *F. culmorum* isolates, Yellow highlight: correlations between epidemics in 2022 and 2023. ^a^ conventional fungicide, ^b^ *organic fungicides*.

**Table 8:** Four-way ANOVA for the fungicide test; deoxynivalenol data with organic and conventional fungicides against FHB in wheat, 2023–2024.

Source of Variance		SQ	df	MQ	F	LSD 5%
A, Fungicide		12,681.1	11	1152.83	17.65 ***	2.36
B, Variety		27,064.6	2	13,532.31	207.29 ***	1.18
C, Inocula		23,757.9	4	5939.48	90.98 ***	1.52
D, Year		6124.1	1	6124.09	93.81 ***	0.96
AxB		7948.3	22	361.29	5.53 ***	
AxC		9279.4	44	210.90	3.23 ***	
AxD		1740.9	11	158.27	2.42 **	
BxC		13,631.2	8	1703.90	26.10 ***	
BxD		12,737.5	2	6368.75	97.56 ***	
CxD		17,912.8	4	4478.20	68.59 ***	
AxBxC		7265.4	88	82.56	1.26 ns	
AxBxD		2445.5	22	111.16	1.70 *	
AxCxD		7562.9	44	171.88	2.63 ***	
BxCxD		6244.5	8	624.45	9.56 *	
AxBxCxD		2748.8	88	24.99	0.38 ns	
Within		47,005.8	720	65.29		
Total		214,978.9	1079			
Significance between main effects and two-way interactions						
Trait	df Main effects	Interaction	df Int.	F	LSD 5%	LSD 0.1%
Fungicide A	11	AxB	22	3.19 *	2.3	3.85
	11	AxC	44	5.45 ***	2.01	3.12
	11	AxD	11	7.27 ***	2.52	5.27
Variety B	2	AxB	2	37.53 *	9.55	37.54
	2	BxC	8	7.95 *	4.46	7.96
	2	BxD	2	2.12 ns	9.55	2.13

*** *p* = 0.001, ** *p* = 0.01, * *p* = 0.05, ns = non-significant.

**Table 9:** FHB mean data of differently resistant wheat varieties across four isolates and two years measured by DON data, mg/kg, 2023–2024.

Fungicide	GK Pilis	GK Szereda	GK Békés	Mean	Sze/Pi	Bé/Pi	Mean/Pi	Variance
^a^ Verben	0.78	1.94	4.98	2.57	2.49	6.38	3.29	4.7
Prosaro	0.60	1.86	7.23	3.23	3.08	11.95	5.34	12.4
Ascra Xpro	0.78	3.25	6.70	3.58	4.15	8.55	4.57	8.8
Queen	0.71	2.16	10.51	4.46	3.04	14.82	6.29	28
Don Q	1.10	5.97	8.50	5.19	5.42	7.72	4.71	14.1
Amistar Xtra	0.46	4.67	11.87	5.67	10.07	25.60	12.22	33.3
*^b^* *Bord. Mixt. + Kén Neo Sc*	1.75	7.67	12.70	7.37	4.38	7.26	4.22	30.1
*Kumulus S*	1.67	8.56	15.92	8.72	5.13	9.54	5.22	50.8
*Polyversum*	2.40	7.29	17.27	8.99	3.04	7.19	3.74	57.4
*Thiovit Jet*	1.74	9.53	16.21	9.16	5.47	9.30	5.26	52.4
*Nevikén Extra 50*	1.87	11.95	20.93	11.58	6.39	11.18	6.19	90.9
Fusarium control.	3.01	9.36	30.09	14.15	3.11	10.01	4.71	200.7
Mean	1.41	6.18	13.58	7.06	4.40	9.65	5.02	
KSD 5% Fungicide	4.09	4.09	4.09	2.96				
LSD 5% variety				1.48				
Correlation	GK Pilis	GK Szereda	GK Békés	Mean	Sze/Pi	Bé/Pi	Mean/Pi	
GK Szereda	0.785							
GK Békés	0.882 ***	0.798 *						
Mean	0.905 ***	0.902 ***	0.979 ***					
Sze/Pi	−0.230	0.284	0.053	0.108				
Bé/Pi	−0.422	−0.179	−0.008	−0.093	0.717 **			
Mean/Pi	−0.390	−0.049	0.011	−0.038	0.849 ***	0.977 ***		

*** *p* = 0.001, ** *p* = 0.01, * *p* = 0.05, ^a^ = conventional, ^b^ = organic fungicide, Sze = Szereda, Pi = Pilis, Bé = Békés. Yellow highlight: correlations between epidemics in 2022 and 2023.

**Table 10:** Fungicide tests against FHB in wheat; DON contamination (mg/kg) for one percentage of disease index (DI) between means across years and isolates, 2023–2024.

Fungicide	GK Békés	GK Pilis	GK Szereda	Mean	Variance
^a^ Verben	0.54	0.17	0.55	0.45	0.047
^*b*^ *Polyversum*	0.59	0.21	0.41	0.46	0.036
Don Q	0.50	0.16	0.68	0.48	0.070
*Bord. Mixt. + Kén Neo Sc 5.0*	0.58	0.18	0.53	0.48	0.048
*Thiovit Jet*	0.68	0.19	0.58	0.55	0.067
Queen	0.76	0.15	0.41	0.56	0.095
Ascra Xpro	0.63	0.22	0.77	0.58	0.081
*Kumulus S*	0.74	0.19	0.75	0.63	0.105
Fusarium contr.	0.96	0.19	0.46	0.63	0.151
*Nevikén Extra*	0.76	0.18	0.71	0.64	0.103
Prosaro	0.84	0.17	0.74	0.66	0.128
Amistar Xtra	0.94	0.07	0.85	0.69	0.226
Mean	0.71	0.17	0.62	0.57	0.096
LSD 5% Fungicide				ns	
LSD 5% Variety				0.09	
Correlations	GK Békés	GK Pilis	GK Szereda	Mean	Variance
Pilis	0.101				
GK Szereda	0.203	−0.308			
Mean	0.881 **	−0.366	0.591 *		
Variance	0.880 ***	−0.693 *	0.506	0.863 ***	

*** *p* = 0.001, ** *p* = 0.01. * *p* = 0.05, ^a^ = conventional, ^b^ = *organic fungicide*.

**Table 11:** Fungicide tests against FHB in wheat; DON contamination (mg/kg) for one percentage of *Fusarium*-damaged kernels (FDKs) between means across years and isolates, 2023–2024.

Fungicides	GK Békés	GK Pilis	GK Szereda	Mean	Variance
*^b^* *Polyversum 0.1*	1.31	1.51	1.20	1.29	0.02
*Thiovit Jet 7.5*	1.62	2.84	1.48	1.61	0.56
*Bord. Mixt. + Kén Neo Sc 5.0*	1.50	3.92	1.73	1.66	1.78
^a^ Don Q 1.0	1.68	2.28	1.67	1.71	0.12
*Nevikén Extra 50*	1.82	2.04	1.90	1.86	0.01
Verben 1.0	1.57	14.06	2.21	1.87	49.49
Queen 1.5	1.97	9.25	1.58	1.97	18.69
Fus. contr.	2.52	1.72	1.22	1.98	0.43
*Kumulus S 4.0*	2.12	6.08	1.72	2.05	5.80
Prosaro 1.0	2.00	13.61	2.65	2.22	42.53
Amistar Xtra	2.67	4.94	2.03	2.49	2.33
Ascra Xpro 1.5	2.51	8.93	2.25	2.56	14.30
Mean	1.94	5.93	1.80	1.94	11.33
LSD 5% Fungicide				ns	
LSD 5% variety				2.01	
Correlations	GK Békés	GK Pilis	GK Szereda	Mean	
GK Pilis	0.883 ***				
GK Szereda	0.245	0.772 **			
Mean	0.880 ***	0.469	0.649 *		
Variance	−0.062	0.954 ***	0.702 *	0.280	

*** *p* = 0.001, ** *p* = 0.01. * *p* = 0.05. ^a^ conventional fungicide, ^b^ *organic fungicide.*

**Table 12:** Fungicides against FHB in wheat; summary table for disease traits across years and varieties, 2023–2024. Percentages of DON mg/kg data for a percentage of DI and FDK. Ranking according DON data.

Fungicide	DI	FDK	DON	Variance	DON/DI	DON/FDK
	%	%	mg/kg		mg/kg	mg/kg
Verben 1.0	5.754	1.372	2.568	5.130	0.446	1.872
Prosaro 1.0	4.867	1.452	3.231	2.916	0.664	2.225
Ascra Xpro 1.5	6.117	1.399	3.577	5.574	0.585	2.556
Queen 1.5	8.006	2.264	4.460	8.393	0.557	1.970
Don Q 1	10.835	3.040	5.190	16.208	0.479	1.707
Amistar Xtra	8.176	2.277	5.666	8.764	0.693	2.489
Bord. Mixt. + Kén Neo Sc 5.0	15.331	4.446	7.372	31.734	0.481	1.658
Kumulus S 4.0	13.904	4.250	8.717	23.342	0.627	2.051
Polyversum 0.1	19.405	6.961	8.988	44.578	0.463	1.291
Thiovit Jet 7.5	16.543	5.694	9.159	30.704	0.554	1.609
Nevikén Extra 50	18.233	6.236	11.583	36.126	0.635	1.857
*Fusarium* control.	22.485	7.130	14.153	59.087	0.629	1.985
Mean	12.47	3.88	7.06	22.71	0.57	1.82
LSD 0.05	1.52	1.20	2.36			
LSD 0.01	2.01	1.58	3.12			
LSD 0.001	2.57	2.02	3.99			
Correlations	DI	FDK	DON	Variance	DON/DI	
FDK	0.9896 ***					
DON	0.9536 ***	0.9374 ***				
Variance	0.9842 ***	0.9673 ***	0.9381 ***			
DON/DI	−0.0554	−0.0465	0.2164	−0.0641		
DON/FDK	−0.5939	−0.6260	−0.3647	−0.5581	0.6960	

*** *p* = 0.001.

**Table 13:** Summary table of the fungicide test of conventional and organic products in winter wheat varieties across 10 epidemic conditions, 2023–2024. Ranking according to mean DON contamination data.

Fungicide	DI%				FDK%				DON mg/kg				
L or kg/ha	GK Békés	GK Pilis	GK Szereda	Mean	GK Békés	GK Pilis	GK Szereda	Mean	GK Békés	GK Pilis	GK Szereda	Mean	Variance
^a^ Verben 1.0 *	9.19	4.54	3.53	**5.75**	3.18	0.06	0.88	**1.37**	4.89	0.63	1.88	**2.47**	6.78
Prosaro 1.0	8.62	3.47	2.52	**4.87**	3.61	0.04	0.70	**1.45**	7.17	0.42	1.78	**3.12**	7.05
Ascra Xpro 1.5	10.67	3.49	4.19	**6.12**	2.66	0.09	1.45	**1.40**	6.03	0.55	3.09	**3.23**	8.63
Queen 1.5 **	13.87	4.89	5.26	**8.01**	5.35	0.08	1.37	**2.26**	10.43	0.66	2.10	**4.40**	17.25
DON Q 1.0	17.02	6.76	8.73	**10.84**	5.06	0.48	3.58	**3.04**	8.42	1.02	5.87	**5.10**	20.82
Amistar Xtra 1.0	12.66	6.36	5.51	**8.18**	4.44	0.09	2.29	**2.28**	11.80	0.37	4.59	**5.58**	16.07
^*b*^ *Bord. Mixt + Kén*	21.85	9.70	14.44	**15.33**	8.46	0.45	4.44	**4.45**	13.50	1.64	8.46	**7.87**	38.71
*Kumulus S 4.0*	21.42	8.92	11.37	**13.90**	7.51	0.27	4.97	**4.25**	16.26	1.55	8.46	**8.76**	37.24
*Polyversum 0.1*	29.06	11.20	17.96	**19.40**	13.20	1.59	6.09	**6.96**	16.62	2.31	7.51	**8.81**	63.32
*Thiovit Jet 7.5*	24.00	9.23	16.40	**16.54**	10.02	0.61	6.45	**5.69**	17.29	1.62	11.00	**9.97**	47.04
*Nevikén Ext. 5.0*	27.53	10.36	16.81	**18.23**	11.50	0.92	6.29	**6.24**	20.89	1.79	12.25	**11.64**	61.65
Fusarium control	31.44	15.83	20.19	**22.49**	11.96	1.75	7.68	**7.13**	30.00	2.92	10.92	**14.61**	94.64
Mean	18.94	7.89	10.58	**12.47**	7.24	0.54	3.85	**3.88**	13.61	1.29	6.49	**7.06**	
LSD5%	Fungicide			1.98				1.59				2.96	
LSD5%	Variety			0.99				0.79				1.48	
	28.4	11.8	15.9	18.7	10.9	0.8	5.8	5.8	20.4	1.9	9.7	10.7	

** *p* = 0.01, * *p* = 0.05. Dark green: lower than 50% of mean; light green: 51–100% of mean; yellow: 101–150% of mean; orange: all above 150%. Bold printed columns: Means for varieties to highlight them, ^a^ conventional fungicide, ^b^ *organic fungicides*.

**Table 14:** Weather conditions for fungicide tests on winter wheat varieties, 2023 and 2024.

Months	T*. °C Middle	T. °C Min.	T °C Max.	Precipitation mm
**2022–2023**				
October 2022	14.8	14.8	22.9	9.5
November 2022	8.4	−0.6	17.8	9.7
December 2022	5	−3.6	15.2	50.8
January 2023	5.7	−1.0	14.8	31.6
February 2023	5	−5.6	18.0	12.7
March 2023	9.8	−0.6	24.1	27.6
April 2023	11.7	0.6	23.4	23.1
May 2023	17.8	6.7	26.9	73.3
June 2023	21.6	11.6	34.4	39
July 2023	25.4	14.2	36.8	36.9
**2023–2024**				
October 2023	16.9	4.7	27.6	18.1
November 2023	8.2	−2.0	18.6	90.6
December 2023	4.7	−4.7	16.7	59.2
January 2024	3.4	−8.0	14.7	21.3
February 2024	10.3	−1.2	20.0	4.6
March 2024	11.4	1.2	26.7	18.0
April 2024	15.6	3.1	30.4	35.4
May 2024	19.4	10.4	28.2	25.7
June 2024	23.9	12.6	35.8	76.0
July 2024	28.0	15.4	38.3	14.1

*T = Temperature; Min. = minimum; Max. = maximum.

**Table 15:** Active ingredients of the fungicides used with the untreated control (UTC) 2023–2024.

Fungicide	Active Ingredient g/L	Prod.	Org/Conv.
Amistar Xtra 1 L/ha	azoxystrobin 200 g/liter + cyproconazole 80 g	Syngenta	a*
Ascra Xpro 1.5 L/ha	prothioconazole 130, bixafen 65, fluidram 65	Bayer AG	a
Bordeaux mixt. + Kén Neo Sc 50 L/ha	CuSO_4_ Bord. complex + 215 sulphur 290 g	FM, Hu	b*
DON Q 1 L/ha	Thiophanate-methyl 500	Sumi Agro	a
Kumulus S 4 L/ha	Sulphur 800	BASF	b
Nevikén Extra 50 L/ha	Polysulphide Sulphur, 58% vaseline oil	Bábolna-Bio, Hu	b
Polyversum 0.1 kg/ha	*Pythium oligandrum*. 1 106 oospore/g	BioGarden	b
Prosaro 1 L/ha	prothioconazole 125, tebuconazole 125	Bayer AG	a
Queen 1.5 L/ha	Fenpikoxamid 50 g + prothioconazole 100	Corteva	a
Thiovit Jet 7.5 L/ha	Sulphur 800 g	Syngenta	b
Verben 1 L/ha	Proquinazid 50 g + prothioconazole 200 g	Corteva	a
*Fusarium* control	no	no	no

a* = organic, b* = conventional.

## Data Availability

The original contributions presented in this study are included in the article material. Further inquiries can be directed to the corresponding author.

## Supplementary Materials

The following supporting information can be downloaded at: https://www.mdpi.com/article/10.3390/toxins18030123/s1, Figure S1: PC analyses for the FB traits, means for 2023 and 2024.

## Abbreviations

mix	Mixture
ANOVA	Analysis of variance
Ctrl	Naturally infected control
DI	Disease index
DON	Deoxynivalenol
Fc	*Fusarium culmorum*
FDK	Fusarium-damaged kernel
Fg	*Fusarium graminearum*
FHB	Fusarium head blight
GK	GabonaKutató, abbr. of the firm name before variety name
HT-2	A-type trichothecene mycotoxin
LSD	Least significant difference
PCA	Principal component analysis
T-2	A-type trichothecene mycotoxin
UTC	Untreated control (natural infection)
VOC	Volatile organic compound
ZEN	Zearalenone
