# Supplementary material for: Stability and Efficacy of Fungicides Registered for Organic and Commercial Wheat Production in Hungary Against Fusarium Head Blight—A Comprehensive Methodology to Enhance Food Safety
Source: Toxins (Basel). 2026 Mar 2;18(3):123. doi: 10.3390/toxins18030123 (PMC13030495; doi:10.3390/toxins18030123)
Supplement: Supplementary file 1 [file toxins-18-00123-s001.zip › toxins-4119384-supplementary.pdf]

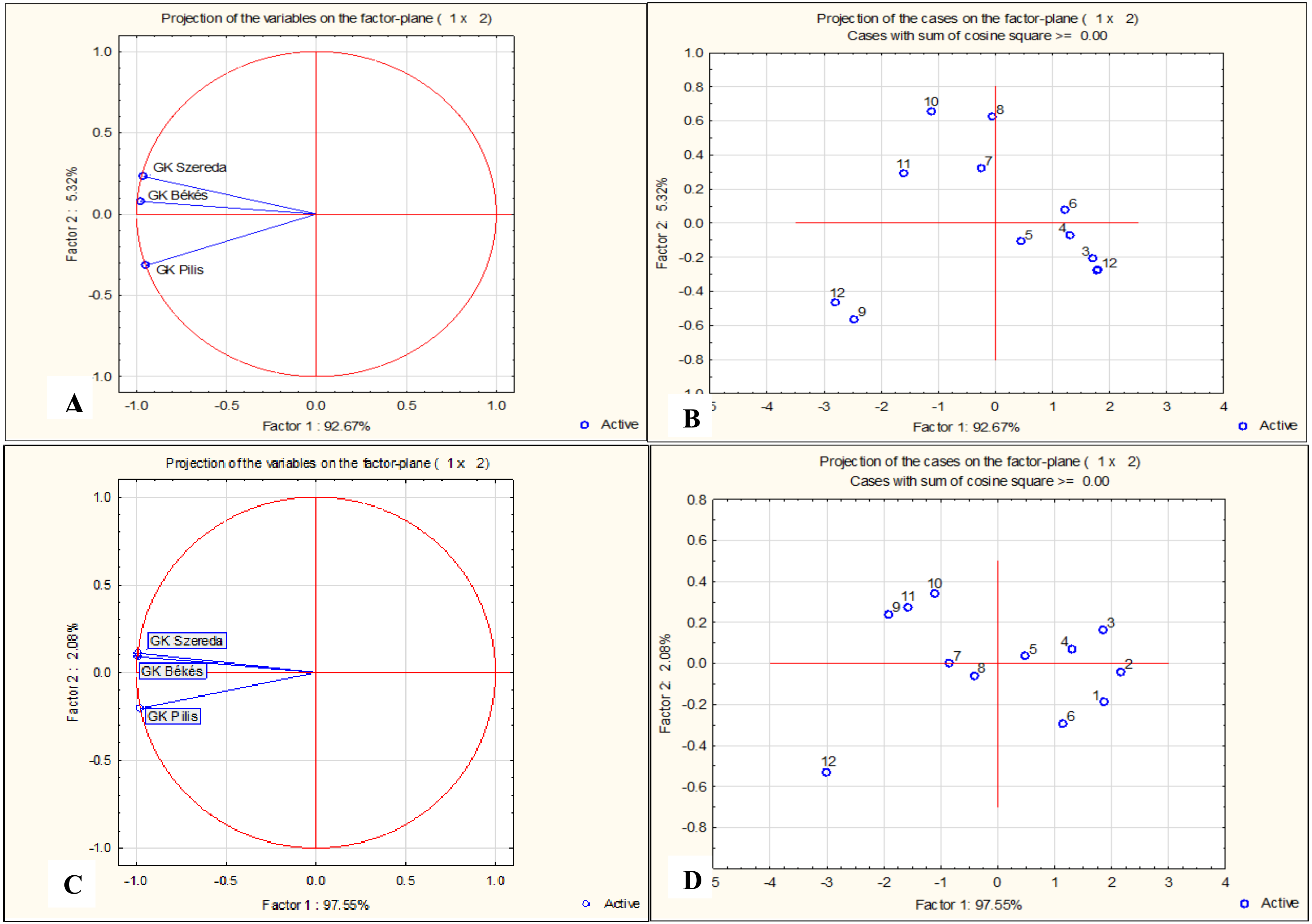

Supp. Figure S1 **A-D**. PC analyses for the FB traits, means for 2023 and 2024. **A**: Disease index for the three cultivars, **B**: DI Fungicide positions, **C**: FDK for varieties, **D**: FDK fungicide positions,

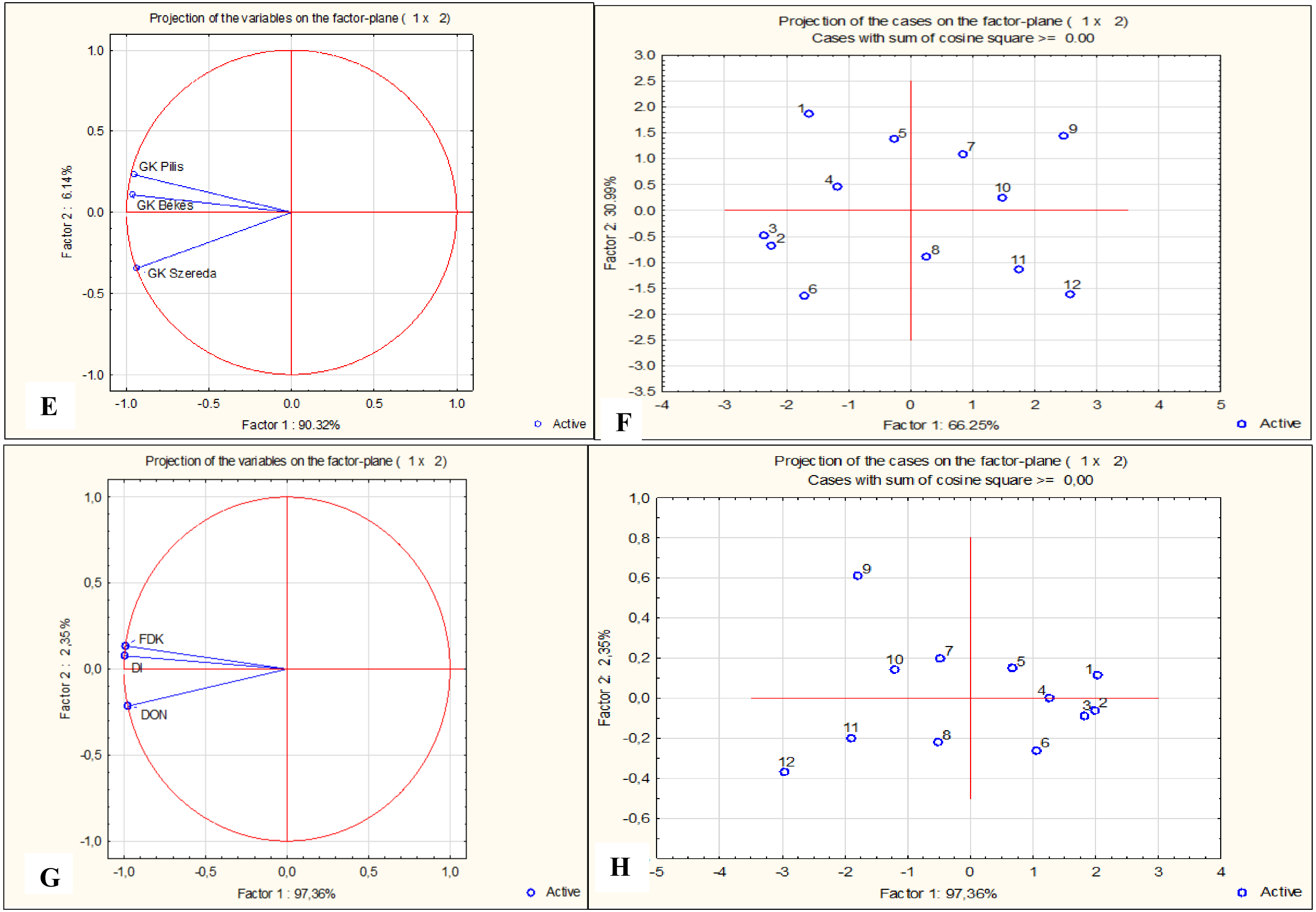

Supp. Figure S1 **E-H**. PC analyses for the FB traits, means for 2023 and 2024. **E**; DON for the three cultivars**, F**: DON; Fungicide positions, **G**: Summary, FDK Traits, **H**: Summary, fungicide positions in co-operation of traits
